# Toward a Common Terminology for the Thalamus

**DOI:** 10.3389/fnana.2018.00114

**Published:** 2019-01-11

**Authors:** Jürgen K. Mai, Milan Majtanik

**Affiliations:** ^1^Institute for Anatomy, Heinrich-Heine-University, Duesseldorf, Germany; ^2^Institute of Informatics, Heinrich-Heine-University, Duesseldorf, Germany

**Keywords:** thalamus, parcellation, nomenclature, terminology, MNI standard space, concordance analysis, human

## Abstract

The wealth of competing parcellations with limited cross-correspondence between atlases of the human thalamus raises problems in a time when the usefulness of neuroanatomical methods is increasingly appreciated for modern computational analyses of the brain. An unequivocal nomenclature is, however, compulsory for the understanding of the organization of the thalamus. This situation cannot be improved by renewed discussion but with implementation of neuroinformatics tools. We adopted a new volumetric approach to characterize the significant subdivisions and determined the relationships between the parcellation schemes of nine most influential atlases of the human thalamus. The volumes of each atlas were 3d-reconstructed and spatially registered to the standard MNI/ICBM2009b reference volume of the Human Brain Atlas in the MNI (Montreal Neurological Institute) space (Mai and Majtanik, 2017). This normalization of the individual thalamus shapes allowed for the comparison of the nuclear regions delineated by the different authors. Quantitative cross-comparisons revealed the extent of predictability of territorial borders for 11 area clusters. In case of discordant parcellations we re-analyzed the underlying histological features and the original descriptions. The final scheme of the spatial organization provided the frame for the selected terms for the subdivisions of the human thalamus using on the (modified) terminology of the Federative International Programme for Anatomical Terminology (FIPAT). Waiving of exact individual definition of regional boundaries in favor of the statistical representation within the open MNI platform provides the common and objective (standardized) ground to achieve concordance between results from different sources (microscopy, imaging etc.).

## Introduction

Modern neuroimaging research requires consistent, internally complete and systematic nomenclature (Swanson, 2015). Particularly for the new generation of discovery tools a solid thalamus parcellation and nomenclature is essential. Traditional textbooks, however, are not helpful as they normally mediate a stereotypical picture of the human thalamus with a spheroid structure in the center of an established standardization grid (standard space) with nuclei that are named according their topographic positions (anterior, central etc.) and show an orderly arrangement of in- and output relations.

In clear contrast to such idealized representations is the complexity of the internal organization and the nomenclature of the human thalamus when it comes to a detailed interpretation. The reader is confronted with multiple parcellation schemes with often bewildering terms. Comparing the different competing delineations and deciphering the innumerable and often non-matching terms is coping with frustration.

The three most important reasons for the disparate and demotivating situation are first, the great individual variations in topographic relationships of human thalamic nuclei, second, the impact of age and disease and, third, the different concepts of researchers from different “schools.”

**Figure 1:**
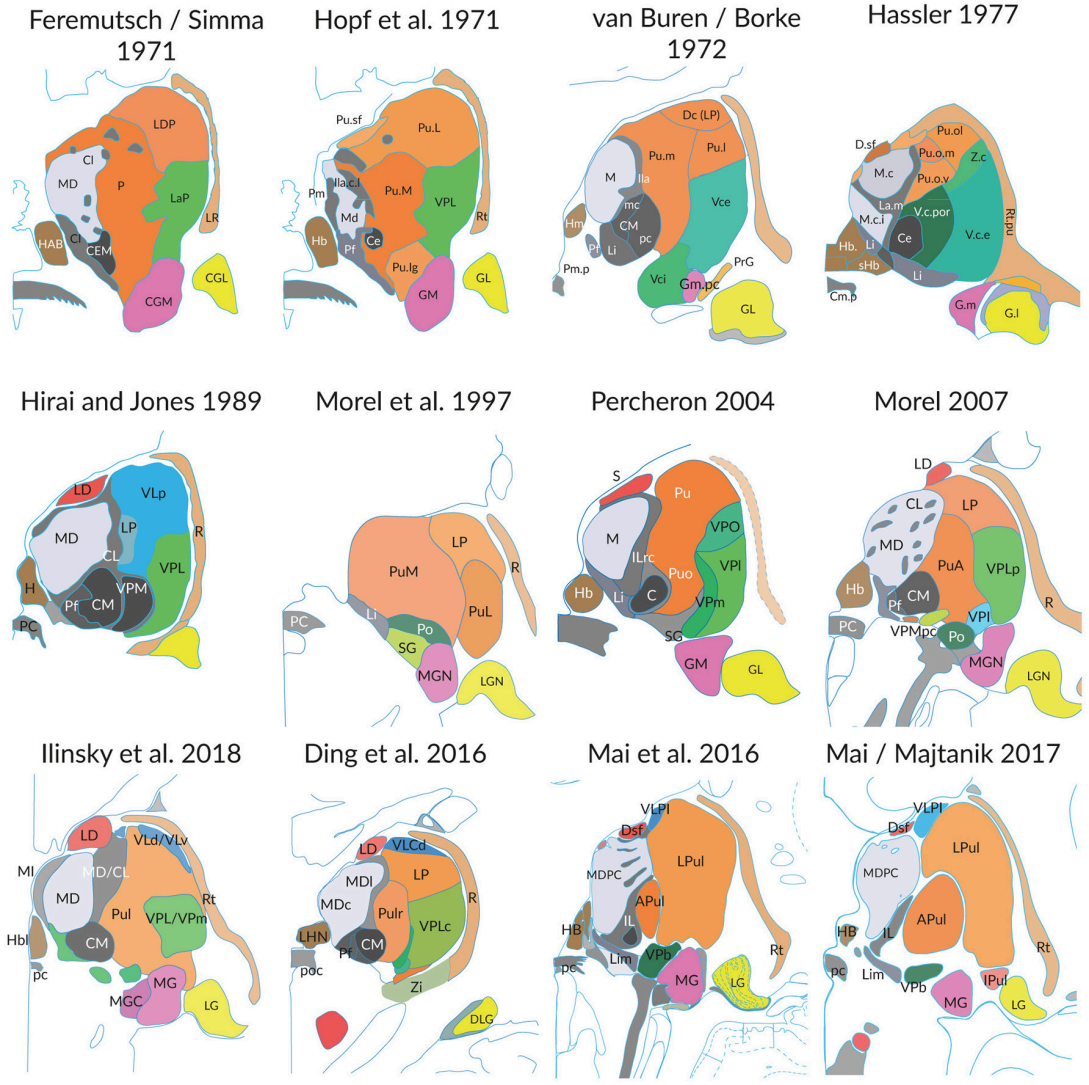
Individual differences of the thalamus in post-mortem brains (medial is to the left). Each figure shows the cross-section areas and topographies of individual thalamic nuclei in coronal sections at the level of the posterior commissure as presented in the original work of the authors. The section planes are close to perpendicular to the intercommisural line (ICL), the reference line connecting the anterior and posterior commissure, except the slices from Hopf, Feremutsch and Simma, Hirai and Jones, Ding. These are tilted up to 22° to the intercommissural plane. The diagrams were redrawn and color was added. The color in each section designates comparable nuclei or territories (blue: cerebellar territory; green: somatosensory complex; orange: pulvinar; light gray: mediodorsal nucleus; dark gray and black: intralaminar nuclei). For abbreviations see Supplementary Table 5.

The extent to which these three aspects influence the representation of the thalamus is illustrated in the following figures. The Figure 1 shows the profile of 12 coronal sections through the thalamus of different brains cut at the level of the posterior commissure. The substantial differences of the profile and the discrepancies of the internal parcellation of the main divisions are obvious, irrespective the different naming of subareas and the different authorships. Similar results are obvious if sections are compared which were cut at any other orientation. The interindividual differences and the great topographic variations hinder to define measures and variables like those advanced for the rodent brain (Swanson and Bota, 2010).

**Figure 2:**
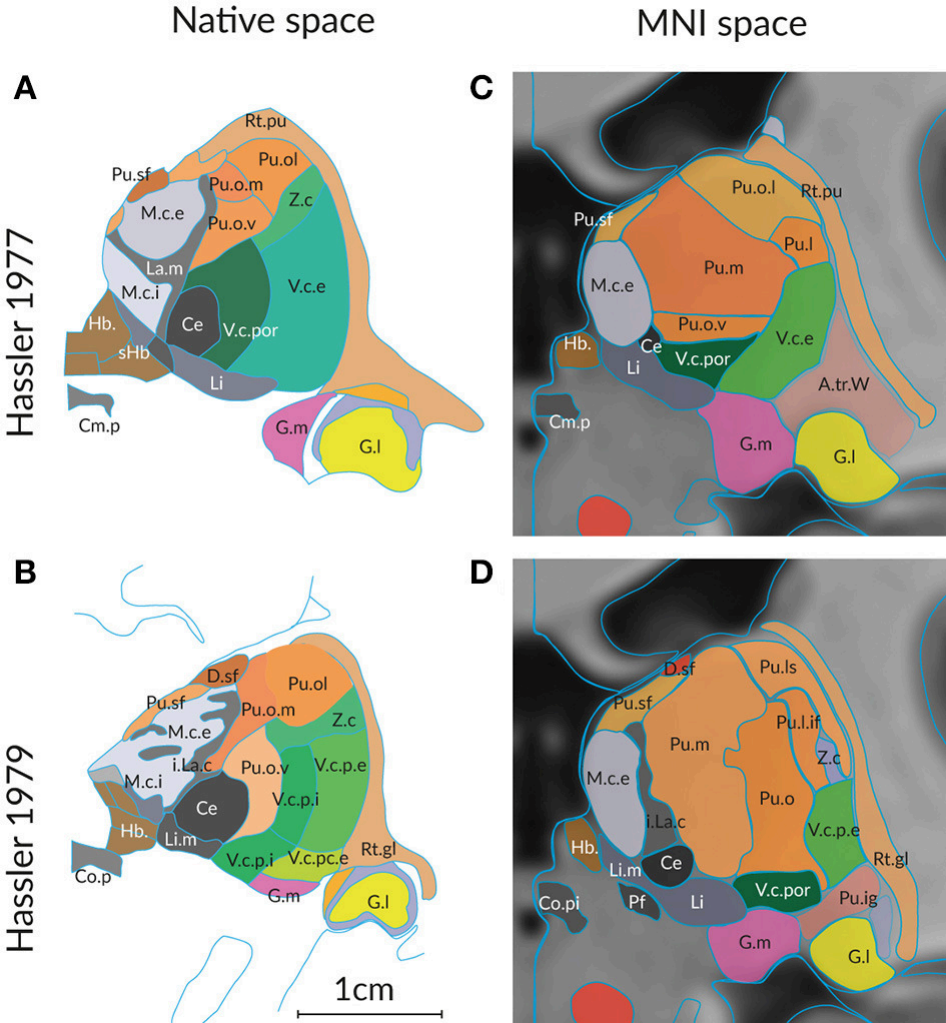
Differences in the structural organization of the thalamus of a “normal” **(A,C)** and a compromised thalamus **(B,D)** analyzed by the same author. Parcellation of the thalamus of a 57 year old male without pathologic change in the brain (Hassler, 1977) and of a person with the diagnosis of Parkinson's disease (Hassler et al., 1979). **(A,B)** Representiation of a coronal section through the posterior commissure as originally published (cf. Figure 1). The scale bar was taken from the original figures; color was added. **(C,D)** Display of the profiles generated from the 3D reconstruction of the serial sections from both cases. The individual 3D volume was registered into the standard space (using the MNI/ICBM co-registered 3D-AHB model) and a coronal section is presented that passes through the posterior commissure. Color code as in Figure 1. For abbreviations see Supplementary Table 5.

The second important reason for differing segmentations of the human thalamus is the influence of age and disease. Global changes in volume, shape and neural connectivity across the adult lifespan are well-studied (Hughes et al., 2012; Hess et al., 2014). Changes of the detailed topographic organization that occur in the course of aging and disease have not been systematically determined with respect to thalamus maps. Figure 2 shows a comparison of the delineations of the thalamus in a “normal” individual and in a case of Parkinson's disease. The delineations by the same author (Hassler, 1977; Hassler et al., 1979) ensure that the same criteria were applied without observer bias. Even under these ideal conditions the maps show clear differences at the corresponding section level. The variance is even more impressive if the delineations are compared on cross sections through the thalamus in the standard space.

**Figure 3:**
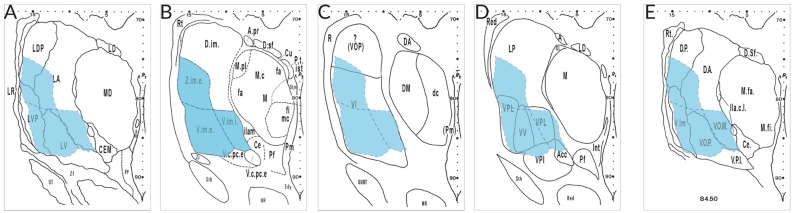
Delineations of the same cell- and fiber-stained sections at mid-thalamic level presented by different specialists. The diagrams were taken from the atlas edited by Dewulf after the Louvain Conference 1963 (publ. 1971) from the following authors: **(A)**, Feremutsch and Simma; **(B)**, Hopf; **(C)**, Macchi; **(D)**, Krieg; **(E)**, Feremutsch and Simma (“standardized nomenclature”). Since all five delineations refer to the same section the V.im region demarcated by Hopf (blue color) was superimposed over the remaining diagrams. The V.im region defined by Hopf (V.im.e, V.im.i, and Z.im.e) is overlapping parts of LDP, LA, LV, and LVP in the diagram of Feremutsch and Simma; it corresponds to the major part of VI but also reaches VOP (Macchi); it is designated as VPL and VV but encroaches also LP, VPI, and Acc (Krieg) and is designated in the “standarized nomenclature” as Vim, VOP and VOM but also includes parts of DP and DA, respectively. Acc, not specified (N. arcuatus); DA, not specified (N. dorsalis anterior); DP, not specified (N. dorsalis posterior); LA, N. lateralis thalami, pars principalis; LDP, N. lateralis thalami, pars dorsalis posterior; LP, N. lateralis posterior; LV, N. lateralis thalami, pars ventralis; LVP, N. lateralis thalami, pars ventralis posterior, V.im.e/i; Ncl. intermedius, pars externa/ interna; VI, Ncl. ventralis intermedius; VOM, not specified (Ncl. ventrolateralis, pars medialis); VOP, Ncl. ventrolateralis, pars posterior; VPI, Ncl. ventralis posterior inferior; VPL, Ncl. ventralis posterior lateralis; VV, N. ventralis ventralis; Z.im,e, Ncl. zentrolateralis intermedius, pars externa.

The most aggravating hindrance for a harmonizing nomenclature is due to different concepts of researchers from different “schools.” Figure 3 gives an example of the interpretation by specialists that were asked to analyze the very same serial thalamus sections. Their maps reveal appreciable differences with respect to architectonic interpretation and terminology. Neither the segmentation nor the terms used for the lateral nuclei show any concordance. The area delineated by Hopf as Ncl. ventro- and *zentro*intermedius (V.im and Z.im.e) that is characterized by well-known cellular features and identified as target for the cerebellar afferents, has almost no areal and conceptual counterpart in the other diagrams. This outcome illustrates that the interpretation of the same cyto- and myeloarchitecture is driven by diverging criteria. This includes the incorporation of certain types of bias and possibly prejudgement depending from experience with animal or human brains, tradition or “schools.”

The regions distinguished in the human thalamus were described with variant terms. They are associative to historical aspects and show linguistic differences (Latin vs. English terminology), were adopted to harmonize the naming system between species and reflect the influence from human pathology. Walker (1966) has properly noted that “… anatomists have attempted to designate thalamic components, delineated morphologically, by topographic or descriptive adjectives, numbers or letters, both Greek and Arabic. Since the compartments so defined have no common point of reference, thalamic nuclei of different schools are not comparable, so the disciples of each creed have adhered rigidly to their own dogma and rejected all others.”

The need of a comprehensible thalamic nomenclature, readable with immediate meaning, has been addressed early. Already 75 years ago Vogt and Vogt (1941/1942) noted in their studies on the human thalamus (“*Thalamusstudien*”) the commitment to rely on areas distinguished by biologically significant features and on the use of an intelligible nomenclature. Their quest for a consistent, derivable naming scheme was also driving the work of many other authors, many of them adding additional parameters from developmental, functional, molecular or comparative studies (Grünthal, 1934; Dekaban, 1953, 1954; Hassler, 1959; Riley, 1960; Andrew and Watkins, 1969; Mehler, 1971; Van Buren and Borke, 1972; Emmers and Tasker, 1975; Hirai and Jones, 1989; Macchi and Jones, 1997; Morel et al., 1997; Jones, 2007; Ding et al., 2016).

In order to keep the varied information manageable and to make it usable for research many tables of synonyms for the human thalamus have been created. Those tables involving studies from authors of different “schools” pretend a suitable comparison but indeed offer a poor basis for the harmonization of the thalamic nomenclature. As can be deduced from Figure 3 they just provide lists of closest matching terms for compartments with limited topographic and semantic congruency.

Another approach to reach “a general agreement … and to establish and to adopt a standardized nomenclature” included the analysis of the very same set of sections through the thalamus by several authorities representing different Anglo-American and German “schools” (Dewulf, 1971). This attempt did likewise not result in a concordant and harmonized nomenclature (Figure 3). The huge discrepancies between the delineations prevented the adoption of the recommended parcellation scheme and the proposed nomenclature.

Instead of deriving at an exemplary or unitary and generally accepted terminology for the human thalamus one faces the emergence of even new parcellations and new terms evoked by the modern imaging and informatics technologies. Explorations of the human thalamus by magnetic resonance imaging (MRI) have provided feature maps that may not match to the anatomically specified nuclei or formations (Keifer et al., 2015; Chien et al., 2017; Kumar et al., 2017). In a time when the usefulness of neuroanatomical methods is increasingly appreciated for modern computational analysis of the brain (Devlin and Poldrack, 2007; Bohland et al., 2009; Mitra, 2014) the limited cross-correspondence between recent anatomical atlases creates fundamental problems.

Considering the strong influences of individual anatomy, the appreciable age- and disease-related changes, the impact of different concepts for the interpretation of thalamic anatomy and the impact of imaging technologies we envision the need for a different approach to resolve some of the inconsistencies in terminology. This approach uses the possibilities offered by computer science and stresses the representation of the variant thalamic areas in a common space. The coordinates in this space represent the communality of any features related to the human thalamus. As a common space we selected the standard MNI/ICBM2009b symmetric template (Fonov et al., 2009). This selection ensures that the coordinates act as unifying concept for the naming of thalamic structures. The objective goal is the negligence of terms in favor of topographic precision. In the end, the terminology which reflects different concepts shall become converted in coordinates which define space.

## Materials and Methods

### Synoptic Representation of Nine Anatomic Atlases of the Human Thalamus

We used nine thalamus atlases (Table 1) represented in two different spaces: the original space and the standard MNI space. The original space refers to the representation of the atlas as in the original publication. The standard MNI space relates to the thalamus space of the MNI/ICBM2009b template.

**Table 1:** Thalamus atlases used in this study.

**Atlas**	**Nr. of cardinal planes used**	**Nr. of areas used**	**Abbreviation**
Hassler, 1977	3	103	HSL
Ilinsky et al., 2018	1	42	ILI
Hassler et al., 1979	1	73	HPD
Van Buren and Borke, 1972	3	53	VBB
Feremutsch and Simma, 1971	1	32	FRM
Percheron, 2004	2	29	PER
Morel, 2007	3	47	MRL
Ding et al., 2016	1	79	DNG
Mai and Majtanik, 2017	1	54	AHB

**Figure 4:**
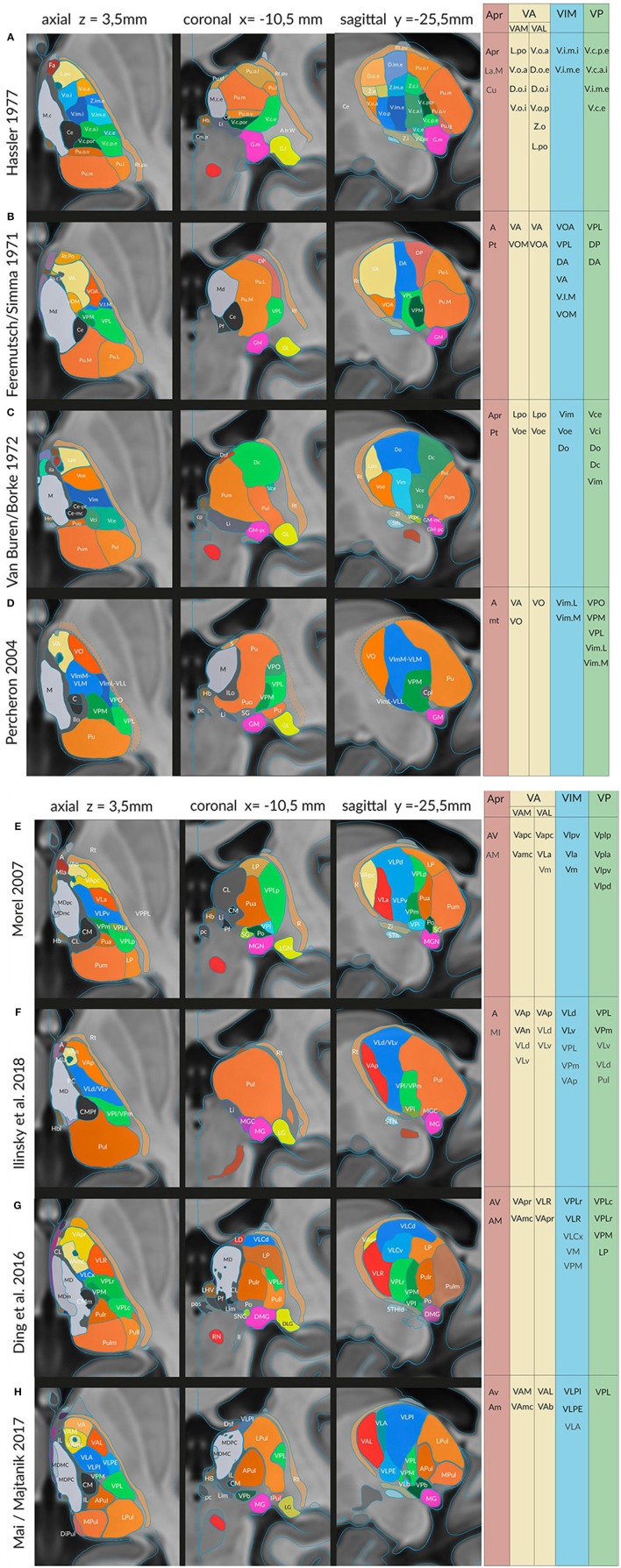
Sections in three cardinal planes from eight different authors indicating segmentations of the thalamus in the common standard (MNI) space: **(A)** Hassler (1977), **(B)** Feremutsch and Simma (1971), **(C)** Van Buren and Borke (1972), **(D)** Percheron (2004), **(E)** Morel (2007), **(F)** Ilinsky et al. (2018), **(G)** Ding et al. (2016) and **(I)** Mai and Majtanik (2017). The exact positions of the three cardinal planes are indicated in the upper line. The color code is the same as in the preceding figures. Right side: areas or nuclei that have been selected for the clusters representing the nigral, pallidal (yellow), cerebellar (blue), sensory (green) territories, and the anterior thalamic nucleus. For abbreviations see Supplementary Table 2.

In the original space only sections or drawings from the publications were used. For the representation of the atlases in the standard space the atlases were 3D reconstructed with our geometric shape constrained 3D reconstruction techniques for serial sections (Mai et al., 2016). Briefly, first a raw model is 3D reconstructed from delineations of one or more histological slice series by simultaneous slice to slice registration procedure. From this model a 3D surface representation is created such that the surfaces are smooth, the surface nodes are distributed according to the inter-slice section distances and the surface area optimally represents the volume of the area. This 3D surface model and its volume representation is then diffeomorphically registered to the MNI space with our procedure (Mai and Majtanik, 2017). The contours of the atlas areas in the MNI space for Figures 2 and 4 were then re-sampled as sections of the 3D surfaces with planes at given locations. Eight of the nine atlases were 3D reconstructed as described and registered to the MNI/ICBM2009b standard space (Fonov et al., 2009). The Ilinsky et al. atlas (Ilinsky et al., 2018) was obtained from the http://www.lead-dbs.org website and registered to the symmetrical MNI/ICBM2009b template.

The “new” atlases representing individual anatomy in the standard space provide a database of parcellation concepts of the human thalamus and of the variant terminology. We have compared the terminology used by the different experts and have listed corresponding regions (Supplementary Table 2). These regions provided the definitions of clusters which were used for the mathematical evaluation of discriminated areas and computation of equivalence of the parcellation concepts. We estimate the equivalence of the concepts by concordance between the areas. We assume that areas with high concordance correspond to equivalent concepts.

Alltogether we have analyzed the following 11 regions: anterior intralaminar region (ILA), central intralaminar region (ILCe), anterodorsal region (A), medial region (M), medial ventroanterior region (VAM), lateral ventroanterior region (VAL), ventrolateral region (VL), ventroposterior complex (VP), posterior region (P), and geniculate region (LGB/MGB). For each cluster we computed concordance of the contained areas across nine thalamic parcellations (Supplementary Tables 1, 2). Not included within the concordance study were the periventricular and midline regions due to the small width of these regions and the inconsistencies in the delineation of the nuclei in the different atlases.

### Concordance Analysis

The degree of conformity of the topography of segmented thalamic regions was assessed by means of concordance analysis. For the concordance analysis an atlas refers to a parcellation of the thalamus in the standard MNI space. The concordance problem can be defined as a quantitative analysis of spatial relationships between parcellations of underlying thalamus space (Bohland et al., 2009). A high concordance of two thalamic parcellations results from high pair-wise spatial overlap between their areas. This analysis provides a valid base for the identification of major conflicts with regard to the characterization and extent of thalamic areas, for comparing the actually used terminology and for defining the most appropriate terms (TNA, 2017). It is understood that we broaden the understanding of “terminology” to include also the anatomic position and neighborhood relations of thalamic structures.

### Hierarchical Analysis Levels

In view of the large quantity of nuclei distinguished by some authors it is appropriate to use a hierarchic scale for the thalamic nuclei. We distinguished between three levels of granularity for the concordance analysis, namely “areas,” “clusters” and “global thalamus.” They denote topographically circumscribed “areas” or groups of structurally and functionally related neighboring areas or “formations” and the sum of all distinguished clusters.

We performed the concordance analysis for these three levels of granularity. The finest, *area* (*local)–level* concordance analysis, compares local pairwise relationship between areas. The middle, *cluster (group)–level* concordance analysis, contrasts 11 clusters and the coarsest, *global (thalamus)–level* concordance analysis, computes concordances between whole thalamus atlases.

For the *area (local)-level concordance analysis*, we analyzed the pair-wise spatial correspondences between anatomical areas defined in the nine different atlases transformed to the ICBM/MNI152_2009b space. For any pair of areas, two conditional probability values were calculated based on the spatial overlap between the areas. Following Bohland et al. (2009) we express the pair-wise spatial relationship as a *conditional probability P(a*_1_*|b*_1_*)* of a voxel being in area **a**_**1**_ according to the atlas **A** if it is in area **b**_**1**_ according to the atlas B. We use a shortened notation for the conditional probability *P(a|b)* = *P*_*ab*_. The results of local area-level concordance analysis show complex correspondences between areas. It is, however, rather difficult to recognize the correspondence between the atlases for specific areas belonging to traditionally defined thalamic subdivisions. To improve understanding and facilitate visualization of the inter-atlases concordance we analyze correspondences between selected groups of areas at the cluster-level.

A *cluster (group)–level concordance measure* should be one if two area clusters from different atlases are perfectly mutually predictable and zero if there is no predictability between the area clusters. Such cluster-level correspondence measure properties are satisfied by adjusted Wallace index (Wallace, 1983; Pinto et al., 2008). The Wallace index W_A→B_ quantifies directional correspondence between two clusters of areas. Given two area groups A and B, Wallace index W_A→B_ between the group A and the group B is the probability that two voxels are classified together in one area in group B knowing that they were classified together in one area in group A. The Wallace coefficient (W) directly indicates the agreement between partitions and therefore can be easily interpreted. As an example, W _A→B_ = 0.832 and W _B→A_ = 0.546 indicate that if two voxel are in the same area in the group A they have about 83% probability of being together in an area in the cluster B, while conversely, this is about 55% probability. This reflects the fact that the group A is more discriminatory than group B and the areas of A subdivide the areas of B.

From the two directional Wallace values we derive the adjusted maximal Wallace index W_max_ = max(W _B→A_, W _A→B_) and adjusted asymmetry Wallace index W_asym_ = abs(W _B→A_−W _A→B_), where max(x) denotes the maximum and abs(x) the absolute value of x.

The scalar-valued maximal Wallace index W_max_ for two parcellations has values between 0 and 1 and emphasizes the highest mutual predictability of two area groups, with one denoting perfect mutual predictability of one area group from the other group. The Wallace asymmetry index W_asym_ takes values between zero and W_max_ and estimates the degree of asymmetry in the mutual predictability of the area groups. A large W_asym_ indicates a strong subset configuration between the two groups. In the above example W_max_ = 0.832 and W_asym_ = 0.286. A *subset* or *subdivision configuration* between areas refers to a spatial relationship where an area from one atlas is divided into multiple smaller areas in other atlas.

*Global (thalamus)–level concordance analysis* estimates correspondences between thalamic parcellations. As a global concordance index we extended the above defined Wallace indices W_max_ and W_asym_ to the whole thalamus parcellations.

Generally three concordance characteristics can be captured by combinations of W_max_ and W_asym_. The first parameter combination with large W_max_ and low W_asym_ indicates high concordance between atlases with predominantly *one to one* relationship of the areas. The second parameter combination with large W_max_ and large W_asym_ points at high concordance of atlases with *one to multiple* area relationships (subset configuration). Finally the third combination with low W_max_ indicates *lack of concordance* between the atlases.

In order to facilitate the interpretation of the Wallace coefficients as being strongly different from the concordance values under chance we estimated concordance distributions for random parcellations of the thalamus volume. We created random partitions of the thalamus volume consisting of N regions with a random label filling algorithm. For each atlas we generated fifty random parcellations with N equal to the number of areas in that atlas. For each pair of atlases the Wallace indices were computed for 1,000 pairs of size-matched random parcellations. The procedure resulted in estimates of the W_max_ and W_asym_ chance distributions specific to each pair-wise atlas comparison. The distributions for cluster concordances were computed analogously. The 95th percentile values (Supplementary Table 4) of these distributions are used to assess whether a given concordance value has <5 percent chance of originating from comparison of random thalamic parcellations.

## Results

### Proposal of a Consolidated Nomenclature: The Base Layer

**Figure 5:**
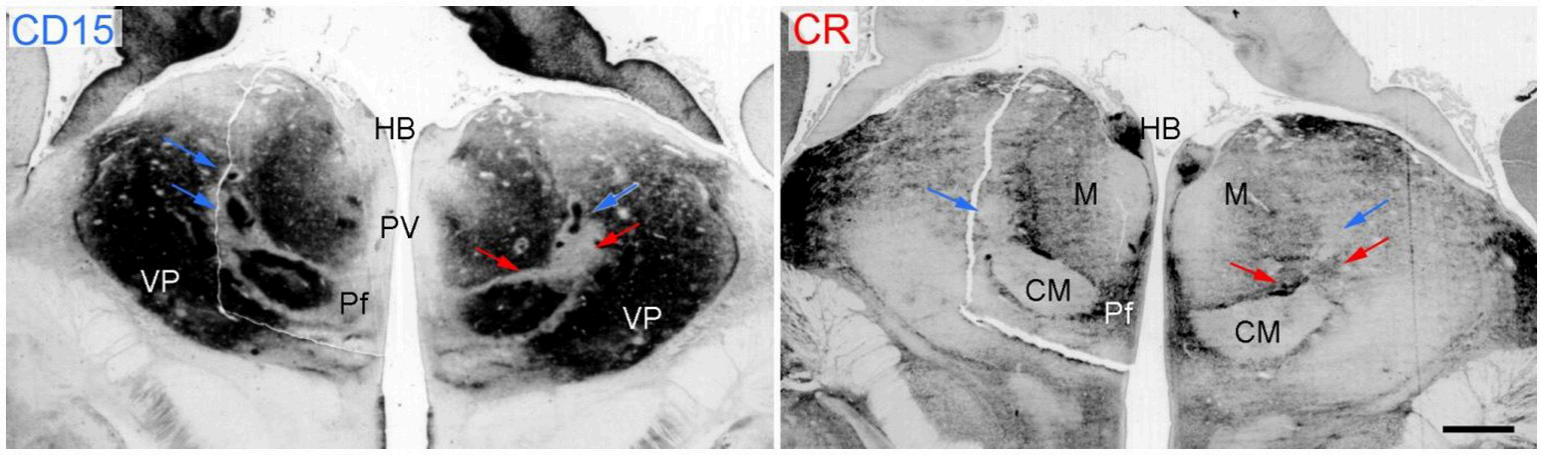
Coronal sections through the thalamus of a fetal human brain at 17 weeks of pregnancy. The immunoreactivity against the cell-surface epitope CD15 (left) and calbindin (right) shows the main thalamic nuclei separated by the intralaminar formation that is CD15 negativ (red arrows) except the associated nuclei (Ncl. centrum medianum CM and Ncl. centralis lateralis, blue arrows) but calbindin positive. Scale bar 1 mm. HB, habenula; Pf, parafascicular nucleus; PV, periventricular region; VP, ventroposterior complex.

The discussion of the nomenclature of the human thalamus concentrates on regions or nuclei which have substantial topographic and functional importance and are suited for the concordance analysis. We begin with the internal medullary lamina and associated nuclei because this extended compartment provides as “great defining landmark” (Jones, 1998) the key for the parcellation and regional analysis of the human thalamus. The relevance for the organization of the thalamus is obvious during prenatal development when it is readily identifiable separating the differentiating neuronal populations of prospective subdivisions (Figure 5; Forutan et al., 2001). Delineation of this formation provides therefore a valuable means for the definition of the topography and the neighborhood relations of thalamic regions.

#### Intralaminar Formation–Formatio Intralaminaris

The intralaminar formation (IL) is represented by a rather dense feltwork of fibers, the internal medullary lamina (Burdach, 1822) or lamella medullaris (Vogt, 1909), that divides the thalamus into medial, lateral and anterior nuclear regions. Embedded within this feltwork are diverse groups of cells that in some locations form circumscribed nuclei (intralaminar nuclei). These cell ensembles have a common developmental history, a characteristic cell type and similar projections to the striatum (see Mai and Forutan, 2012). Two populations are identified histochemically either by calbindin and CD15 or calretinin immunoreactivity.

**Figure 6:**
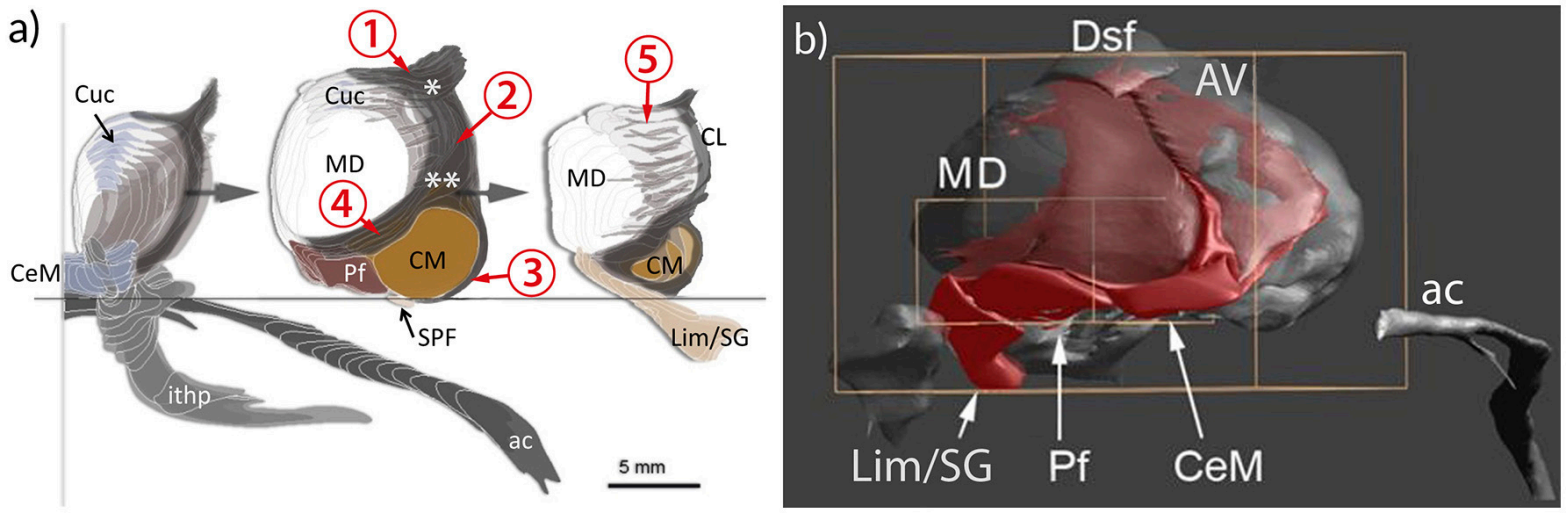
Representation of the intralaminar formation (IL) in relation to the mediodorsal nucleus (MD) and the anterior commissure (ac) and the inferior thalamic peduncle (ithp) as orientation marks. **(a)** MD is sliced to show the anterior, middle and posterior divisions of IL separately. The core of the formation is represented by the portion nestling around the lateral perimeter of the MD and separating the medial from the lateral region of the thalamus. This central portion bifurcates anterodorsally (asterisk) to form the shell below the anterodorsal region. Ventrally the internal lamina also bifurcates (double asterisk) to form a cap that bounds the centromedian nucleus (CM) and then continues around the anterior pole of MD. Cells within the branching areas are the central lateral nucleus (asterisk) and the paracentral nucleus (double asterisk). At the anterior and posterior pole of the MD the internal medullary lamina enlarges and differentiates as central medial nucleus (CeM) anteriorly and as suprageniculate and limitans nuclei (Lim/SG), respectively. **(b)** Medial view of a reconstruction of the IL (red). The IL extends along the ventral surface of MD, represented by the central medial nucleus (CeM), parafascicular nucleus (Pf) and Lim/SG. 1–5: divisions of the IL; 1, superior part; 2. central (lateral) part; 3, circumcentral part (lamella intermedia, Schnopfhagen, 1877; lamella praesemilunaris; Hassler, 1982); 4, anterior part; 5, posterior part (retrocentral part); Cuc, cucullar nucleus.

The extent and the arrangement of cells and fibers of the internal medullary lamina have been characterized differently. The nuclei associated with this lamina were allocated by most authors to an anterior and posterior division. The anterior portion (Ncll. intralaminares anteriores) is represented by the central medial, paracentral, central lateral, and the cucullar nuclei (Hassler, 1959); the posterior portion consists of the Ncl. centrum medianum (centre médian) and parafascicular nuclei (CM/PF) and the subparafascicular nucleus (SPF). However, many authors also agree that the internal medullary lamina continues beyond CM/PF to the pretectal area (Grünthal, 1934; Feremutsch and Simma, 1954a,b; Hassler, 1959; Percheron, 2004; Jones, 2007; Lenz et al., 2010). This posterior division (Ncll. intralaminares posteriores) shows no clear border and the cell ensembles therein seem to interlock with the mediodorsal nucleus (MD). The associated nuclei are notably the limitans, suprageniculate and posterior nuclei (the posterior nuclear complex) and possibly also the pregeniculate nucleus and the magnocellular division of the medial geniculate body (Lenz et al., 2010). The three components of the IL were integrated within the intralaminar-limitans-retrocentral formation (Percheron, 2004) or involucrum (Hassler, 1959) and are now distinguished as the anterior, central and posterior group of the human intralaminar nuclei (Mai and Forutan, 2012; TNA, 2017; Figure 6).

We have applied the group-level concordance analysis separately for the anterior and central division of the intralaminar nuclei. The maximal Wallace index for the anterior division is W_max_ = 0.54 and the asymmetry Wallace index is W_asym_ = 0.16 and for the central division W_max_ = 0.68 and W_asym_ = 0.27 (Table 2A). These values indicate very low concordance with undetermined subdivision relationships between these areas from different atlases. The very low concordance for IL derives from the highly variable representation of IL in the different atlases. For example, Feremutsch and Simma (1971) did not delineate a continuous IL but isolated segments. In contrast, Morel (2007) defined the posterior portion of the IL five times thicker than Mai et al. (2016) or Ding et al. (2016). Such great differences make a re-evaluation of the IL mandatory.

**Table 2A:** Cluster-level concordance.

	**Clusters**									
	**IL**	**AN**	**CM/PF**	**MD**	**VAM**	**VAL**	**VL**	**VP**	**P**	**LGB/MGB**
Concordance Index W_max_	0.55	0.71	0.69	0.85	0.79	0.61	0.71	0.71	0.80	0.84
Asymmetry Index W_asym_	(0.16)	0.24	0.27	0.35	0.27	(0.22)	0.25	0.29	0.27	0.32

*The table displays the results using the maximal Wallace index W_max_ and asymmetry Wallace index W_asym_ computed for nine clusters as a measure for inter-atlas group concordances and subset relations of the areas within the groups*.

#### Periventricular and Midline Region

The region between the ependyma of the third ventricle and the MD is relatively thin in the human thalamus if compared with the corresponding region in subhuman thalami. We distinguish two components: First, the thin sheet of small neurons along the third ventricle below the ependyma which is a component of the ventricular gray substance (Nuclei para- or subependymales thalami, Riley, 1960; substantia grisea centralis thalamica, Hassler, 1982). The second and main portion is constituted by clusters of cells located laterally from the subependymal gray layer. They are collectively termed as midline nuclei knowing that they are defined differently in the literature. We distinguish the paratenial and paraventricular nuclei as the dorsal component, and the reuniens, submedius, and fasciculosus nuclei as the ventral component. The midline nuclei are architectonically strikingly distinct and have different connections. This has let to variant interpretations of their functional relations (Benarroch, 2008).

We address this region because of the considerable differences between the human and subhuman organization and the dissenting opinions about the relation between the cell groups of this region with those of the intralaminar formation. It has been described under several names: midline nuclei (Altman and Bayer, 1988; Krauth et al., 2010), “mediane Kerngruppe” (Niimi, 1949), subependymal formation or paraventricular formation (in Dewulf, 1971; Dom, 1976), midline and epithalamic region (Van Buren and Borke, 1972), paramedian formation (Percheron, 2004). None of these terms refers only to the subependymal or periventricular site of the third ventricle but includes also adjacent areas (Rose, 1942; Van Buren and Borke, 1972; Morel et al., 1997; Krauth et al., 2010; Ding et al., 2016).

#### Anterodorsal Region

The anterodorsal region forms an oblong rostrocaudally oriented structure that extends from the anterior pole to the dorsal (upper) surface of the thalamus. The entire region is separated from the lateral ventricle by a prominent fibrous layer, stratum zonale, and underlaid by the lamina medullaris superior, the superior bifurcation of the internal medullary lamina (Figure 6). It consists of the anterior nuclei and the dorsal superficial nucleus. The latter nucleus is included due to architectonic and hodologic commonalities.

The *anterior nuclei* consist of the “principal” anteroventral nucleus (AV), underneath the anterior tubercle and of the anteromedial (AM), and anterodorsal (AD) nuclei. The dorsal component of the anterodorsal region is represented by the dorsal superficial (or laterodorsal) nucleus. It appears as flat elongation of the anteroventral nucleus approximately up to the middle of the rostrocaudal dimension of the thalamus. Both parts can be distinguished thanks to the fragmentation of the surrounding medullary fibers.

The AV (Sheps, 1945) is named in analogy to subhumans; anterior principal nucleus (Ncl. anteroprincipalis, Vogt and Vogt, 1941/1942) would be a more appropriate term matching its dominant size in humans. The remaining anterior nuclei were often regarded as accessory, aberrant or even as non-existent. They are, however, distinguished by their individual neurochemical characteristics (see Forutan and Mai, 2012). AM appears as extension of AV toward the frontal pole of the thalamus and bends medially to come close to the midline at the interthalamic adhesion. The distinction of one or even multiple interanteroinferior nuclei (Ncl. anteroinferior, Ncl. anteroreuniens, Hassler, 1982, or Ncl. interanteromedialis, Rioch, 1929) for the most medial division next to the ventricular surface is not justified since the anterior nuclei are not merged at the midline in humans. AD is reduced to a small slot-like ensemble of cells between AV and paratenial nucleus. The distinction between the nuclei of the anterodorsal region is relevant because of the structural and functional segregation of the entrant pathways from the extended hippocampal formation and different projections to the cingulate cortex (see Bubb et al., 2017).

The group-level concordance analysis resulted in the maximal Wallace index being W_max_ = 0.71 and the asymmetry W_asym_ = 0.24 (Table 2A). The values indicate relatively good concordance in this cluster with frequent subdivision relationship between the areas from different atlases. The evaluation shows good overlap at the core area of AV-region but rather variations in the DSf region. The high W_asym_ stresses that some authors did not distinguish the various subdivisions of the anterior nuclei. A more detailed interpretation of the subdivisional relationships between these nuclei is illustrated in Figure 9B. The atlases MRL, DNG, HSL and AHB subdivide the anterior ventral nuclei into two or more areas, whereas the remaining atlases delineate only one area.

#### Medial Region (Mediodorsal Nucleus)

The medial region comprises the field encircled by the IL and midline nuclei. It extends from the interthalamic adhesion to the level of the posterior commissure (and thus covers about 2/3 of the total length of the thalamus). In humans this region coincides with mediodorsal nucleus. This definition stresses the distinctiveness against the IL and the midline nuclei which both show relevant developmental, cytological and chemical differences (Forutan et al., 2001). MD is not a homogeneous nucleus as described by Andrew and Watkins (1969). Even less justified is the fragmentation into six or more subnuclei (Namba, 1958; Hassler, 1959; Gihr, 1964; Niimi and Kuwahara, 1973; Ding et al., 2016). It is common to distinguish three major internal divisions: medial, central and paralaminar (TNA, 2017). Other designations used are determined by either the preference for cyto-, myeloarchitectonic or pure topographic criteria. Based on myeloarchitectonic criteria these are pars fibrosa, fasciculosa and paralamellaris (Hassler, 1959); the largely congruent cytoarchitectonic divisions are the magno-, parvo- and densocellular (or multiform) divisions (Olszewski, 1952; Ding et al., 2016). The terms magno- and parvocellularis for the medial and central divisions may be questioned because morphometry does not support the distinction by cell size in humans (Dewulf, 1971; Van Buren and Borke, 1972). A medial subregion, clearly identified by neurofilament-, CART (cocaine- and amphetamine-regulated transcript)- and also CD15-immunoreactivity, may correspond to the territory of amygdaloid afferents (Forutan et al., 2001). The lateral and posterior periphery of the MD along the laminar border is poorly determined. This creates a wide transitional area that extends over this lamina into the pulvinar. The corrugated pattern affiliates this area to either MD or IL which results in either an extended definition of the MD (Mai et al., 2016) or of the IL (Morel et al., 1997). Gihr (1964) described “giant cells” as specific for this paralaminar or transitory division in humans.

The designation “mediodorsal” nucleus is maintained albeit the homolog, the medioventral nucleus, of humans is not part of the medial region but corresponds to a component of the anterior division of IL: the reuniens nucleus and possibly the submedial nucleus.

The term dorsomedial nucleus is inappropriate; it does not describe the topographically correct location within the thalamus and its counterpart would then be consequently described as ventromedial nucleus, a name reserved for the lateral thalamic region.

The maximal Wallace index is W_max_ = 0.85 and the asymmetry Wallace index is W_asym_ = 0.35. These values indicate the highest concordance between the atlases with very strong subdivision relationships between the areas delineated in different atlases. This subdivision configuration of the M cluster is reflected in the conditional probability values in the Supplementary Table 3. The M cluster data show a consistent dominant overlapping of one area accompanied by one or two areas with minor overlap (MRL, ILI, DNG). The spatial distribution of the high concordance coincides with the extend of the MD areas (**Figure 11**, upper row). Similarly, the strong asymmetry W_asym_ is bounded to the extension of the MD area.

#### Lateral Region

The lateral region is defined as the area between the internal medullary lamina medially, the external medullary lamina (lamella perithalamica), reticular nucleus and internal capsule laterally and the posterior region (pulvinar) posteriorly. The territory is well outlined especially on axial sections at midlevel of the thalamus. Functionally, the nuclei of the lateral region are identified and characterized by the target/source of their afferent/efferent projections. The *motor* thalamus receives predominatly (indirect) striatal (nigral and pallidal) and cerebellar (and vestibular) input whereas the *sensory* thalamus receives somesthetic and visceral input (Vogt, 1909; see Percheron, 2004). The exact definition of the territories, the afferent fibers and their relation to the projection neurons in humans awaits still clarification (Jones, 2007; Kaas, 2012). Figure 4 illustrates that the parcellation, based mainly on histological and histochemical methods, is still very controversial and renders this region a very problematic place in terms of nomenclature.

The main divisions were already specified in the cercopithecan brain by C. Vogt (1909). She distinguished between lenticular (pallidal), prelemniscal (cerebellar) and lemniscal radiations terminating in the ventral oral, intermediate (intermédiaire) and caudal division, respectively. These targets correspond to the ventroanterior (VA), ventrolateral (VL or ventral intermediate, V.im) and ventroposterior (VP) nuclei of later authors (Walker, 1938). With the definition of the territory for the afferents from the substantia nigra (SNR) in the rostralmost part of the lateral thalamus (see Ilinsky et al., 2018) this clear terminology became complicated (Supplementary Table 2). The documentation of additional afferents to the lateral region (amygdaloid, kinesthetic, and other fibers) has further impaired the development of a unified terminology.

Other important challenges which influence the segmentation and the terminology of the nuclei of the lateral region were reviewed by Percheron (2004). Most important are geometrical particularities due to the strongly curved main axis and the obliquely arranged nuclei along this axis. The geometric deformation displays the lateral nuclei in cardinal sections as if overlaying each other. If, for example, coronal sections are made, the adjacent nuclei may be cut obliquely depicting alternating volumes. The partial volume effect provokes the questionable distinction between ventral and dorsal partitions of the lateral region. That distinction dates back to Meynert (1872) and had functional impact. It should signify the difference between ventral (V) nuclei that receive “fibers of extrathalamic construction, while symbol D signifies that the nucleus receives no afferent extrathalamic fibers” (Hassler, 1971). Such differentiation between relay and associative nuclei in the lateral thalamus has fundamentally influenced the terminology of the nuclei of the lateral region (Sheps, 1945; Hassler, 1959, 1977; Hopf et al., 1971; Mehler, 1971; Van Buren and Borke, 1972; Niimi and Kuwahara, 1973; Ding et al., 2016). The separation of ventral, dorsal and even central sections of the motor thalamus is unjustified “because the three territories and the individual axons extend over the entire ventrodorsal extent” (Percheron, 2004). Hassler has dropped the “*zentralis*” divisions (Hassler, 1971) but these areas remained delineated in later versions of his atlas diagrams (Hassler, 1977; Hassler et al., 1979). Because the distinction between ventral and dorsal divisions is no longer relevant it would be consequent to label the nuclei within the region lateral to the internal medullary lamina as “lateral nuclei of the thalamus” (Grünthal, 1934; Feremutsch, 1963; Percheron, 1997; TNA). This is, however, unlikely because it has no connection to experimental studies and recent history (Jones, 1997a).

**Figure 7:**
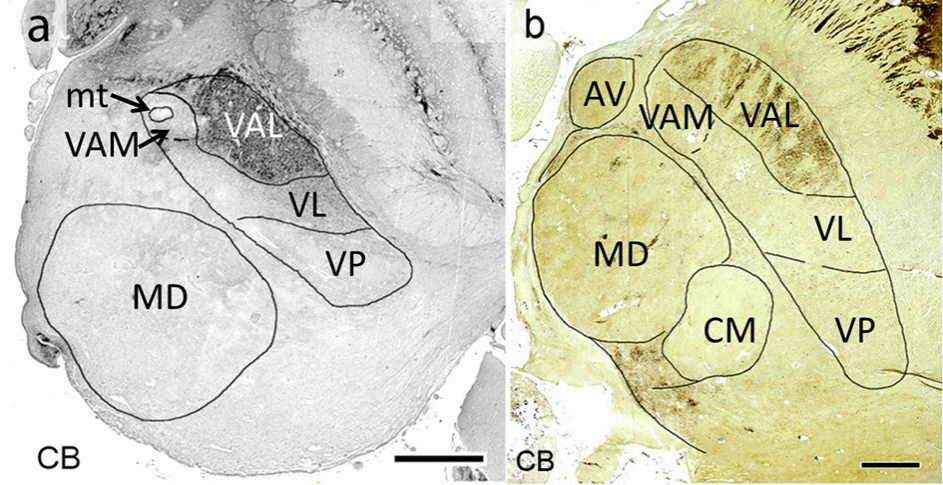
Horizontal calbindin-stained sections through the middle of the mediodorsal nucleus at 19 weeks of gestation **(a)** and at adulthood **(b)**. Within the lateral nuclei calbindin-immunoreactivity is observed exclusively in the pallidal territory (VAL).

Disagreements also exist with regard to the extent to which the territories from different afferent fiber systems overlap. This issue is important because it determines how accurate the borders between the various motor and sensory territories can be drawn. Earlier investigations, including post-mortem studies, indicated that both striatal (nigral and pallidal) fiber systems have well-defined areas of convergence with the cerebellar territory (Mehler, 1971). Recent anatomical studies employing tracing techniques as well as electrophysiological evidence have indicated segregated but interdigitating territories in subhuman primates (see Hintzen et al., 2018). That this organizational principle may also be valid for the VA-VL-VP limits in the human brain is indicated by the clear cytoarchitectonic borders (see Jones, 1998, Figure 9A and Lenz et al., 2010, Figure 2.23a), by interdigitation of fringes described by Percheron (2004) and by the clear contrast between the calbindin-positive VA and the very moderate intensity level in VL (Figure 7).

Another hindrance is posed by discrepancy between Anglo-American authors that are rather “lumpers” while German authors are the so-called “splitters” (Dom, 1976) referring to which extent the lateral nuclei are segmented into subnuclei. Hassler (1977) distinguished excessively high numbers of subdivisions (19 divisions in the motor nuclei of the lateral region) whereas other researchers identified only few divisions in the same area. The correspondence between those multiple areas is rather ambiguous and even questionable if there is no difference in their connectivity and functions, making “some rationalization” (Jones, 1985, p. 378) worthwile.

In our analysis the “splitters” and “lumpers” can be identified by a combination of the Wallace based indices W_max_ and W_asym_. One can observe high W_max_ and low W_asym_ within the both groups and high W_max_ and high W_asym_ between the groups. For example, the comparison of “splitters” (HSL, Hassler, 1977) and (DNG, Ding et al., 2016) shows high concordance W_max_ = 0.71 and very low asymmetry W_asym_ = 0.01. On the other hand, the comparison between “splitters” and “lumpers” (HSL and PER, Percheron, 2004) shows high concordance W_max_ = 0.83 and high asymmetry W_asym_ = 0.69 values. This difference can also be seen in the Supplementary Table 3 for cluster conditional probabilities. The atlases of “splitters” (HSL,HPD) overlap with the clusters in more areas compared to the atlases of “lumpers” (PER).

##### Motor thalamus

The interpretation of the organization and delineation of the areas related to the motor thalamus were a matter of intense dispute (see Percheron et al., 1993). Hassler, Hirai and Jones and Morel et al. divided the pallidal projection field into an anterior and posterior division. These areas were described as V.o.a and V.o.p by Hassler (1977) and as VApr and VLa by Hirai and Jones (1989) and Morel et al. (1997) (see Supplementary Table 2). This may be an unnecessary complication because there are presently no obvious cytological, hodological or histochemical differences between both divisions (Ohye, 1990; Münkle et al., 2000; Forutan et al., 2001). Even more questionable is the partitioning by Hassler (1959) who subdivided the territory which now appears to correspond to the target fields of the nigral and pallidal afferents into more than 10 subnuclei: Ncl. latero-polaris with five subdivisions, Ncl. fasciculosus and Nuclei ventro-orales, Ncl. *zentro*lateralis and Ncll. dorso-orales (see Percheron et al., 1996). He regarded the anterior part of the ventro-oral nucleus (V.o.a) as a terminal area of pallidal, the posterior part (V.o.p) as a terminal area of cerebellar fibers.

Given the limited knowledge about the organization of the motor thalamus in humans it appears reasonable to subdivide it into only three territories which serve as targets for the nigral, pallidal and cerebellar afferents. These are the ventral anterior nucleus with medial and lateral divisions and the ventral lateral nucleus or complex (TNA, 2017).

The *ventral anterior nucleus* (VA; TNA) provides the target for basal ganglia afferents (from the internal pallidum and substantia nigra pars reticulata, respectively) (Ilinsky and Kultas-Ilinsky, 2001). They occupy the anterior pole of the lateral nuclei. The fibers from both sources have been described to branch in rather separate medial and lateral areas.

Afferents from the substantia nigra pars reticulata terminate in the medial part of VA in an area around the mammillothalamic tract, named according to its position in the lateral region as medial ventroanterior nucleus (VAM). This part contains large neurons, a characteristic feature which led to the alternative designation as magnocellular ventroanterior nucleus (TNA). The remaining largely lateral region is the territory for the fibers from the internal pallidum. It is named the principal division (TNA) or in correspondence to the mediolateral arrangement of both striatal territories the lateral ventral anterior nucleus (VAL, Mai et al., 2016). Ilinsky et al. (2018) used the main sources of the afferent fibers to designate both regions of VA (VAn–nigral region; VAp–pallidal region). This might be too restrictive as the medial region also receives afferents from the amygdala and limbic cortex. The attribution of a common name (VA) for the (at least) two target areas (VAM and VAL) appears justified because the afferents derive from the GPi/SNR-complex which was split during development by the fibers of the internal capsule. Both projections use GABA as transmitter. Their territories can, however, be separated by their different developmental timeline with respect to synaptogenesis (Kultas-Ilinsky et al., 2003), their different projection to cortical areas without overlap (Percheron, 2004) and their chemoarchitecture because the nigral (medial) VAM (VAmc) area is calbindin negative (sometimes weakly positive) whereas the pallidal (lateral) area VAL is calbindin positive (Morel et al., 1997; Forutan et al., 2001; Calzavara et al., 2005) (Figure 7).

The *entry zone* of the nigral afferents is poorly defined. The loosely arranged fibers enter VAM (VAmc) above the anterior field of Forel. This area corresponds to the Ncl. lateropolaris basalis (L.po.b) of Hassler and probably to the principal medial nucleus (Jones, 1985, p. 384). The pallidal afferents invade the thalamus as a compact fiber bundle, the thalamic fascicle (h1). Their entrance zone was described by histological evidence in the human brain as the anterior part of the ventromedial nucleus (VM) Gallay et al. (2008). Mai and Forutan (2012) who mapped the succession of prethalamic fibers from the substantia nigra, the internal pallidum, the cerebellum, the spinal cord and the brain stem on histological sections described the entrance zones for the fibers as basal subnuclei of the respective territories. They were accordingly named as basal ventral anterior nucleus (VAb, with VAMb and VALb subnuclei), as basal ventral lateral nucleus (VLb) and the basal ventral posterior nucleus (VPb) (Mai and Forutan, 2012, Figure 19.24).

The maximal Wallace index for VAM (nigral region) reads W_max_ = 0.80 and the asymmetry Wallace index is W_asym_ = 0.27. These values indicate *strong concordance* between the atlases with frequent subdivision relationships between the areas from different atlases. The indices for the VAL (pallidal region) W_max_ = 0.61 and W_asym_ = 0.22 suggest *high variability* between the atlases (unrelated to the terminology).

The *ventral lateral nucleus* (or complex; VL, TNA) provides the target for fibers from the deep cerebellar nuclei but also from the vestibular system and possibly from some kinesthetic neurons. It occupies the area between VA and VP and corresponds to the ventral intermediate nucleus (V.im, Ncl. ventralis intermedius in the terminology of C. Vogt, 1909, as the target of the prelemniscal radiation). V.im was also used by Crouch (1934), Hassler (1959), and Percheron (2004) whereas Jones (2007) and Morel et al. (1997) followed the terminology of Walker (1938) with ventral lateral posterior nucleus (VLp). VLp, however, appears as an inappropriate term because two anatomically, histochemically and functionally different components, one (VLa) receiving pallidal, the other (VLp) cerebellar afferents, are regarded as components of the same VL-region. Nieuwenhuys et al. (2008) described a third component (VLm) receiving nigral afferents.

With the acetylcholesterase (AChE) reaction the VL region is weakly labeled (Hirai and Jones, 1989; Lenz et al., 2010). This contrasts with the very high intensity of VAL (VLa, Lenz et al., 2010, p. 116). Parvalbumin-immunoreactivity also specifies the cerebellar area (Percheron, 2004, p. 627).

VL is divided for topographic reasons in anterior and posterior subdivisions (VLa and VLp; TNA, 2017) which correspond to areas described by Hassler (1959, 1977) and Percheron (2004) as medial and lateral subdivisions. The posterior ventrolateral subdivision (VLp) is clinically relevant because this corresponds to the so-called ventrointermediate or “VIM area” where “tremoro-synchronous” neurons were localized (Albe-Fessard et al., 1966; Ohye and Narabayashi, 1979). Their location coincides with the target selected for the management of some motor symptoms in movement disorders (Ohye, 1990). Mapping the coordinates of contacts accountable for clinical improvement to the standard atlas Mai and Majtanik (2017) shows the location distal to V.im either in the area of the cerebello-rubro-thalamic fibers shortly before entering the thalamus (close to the posterior subthalamic area) or within the entry zone of the fibers which corresponds to the basal ventrolateral nucleus (VLb) (Fiechter et al., 2017; Figure 12; see discussion).

The cerebellar afferents take a position parallel to the ventral thalamic lamina, just ventral to the subparafascicular nucleus and anterolaterally to the parvocellular part of the ventral posterior medial nucleus (VPPC / VPMpc) before they enter the thalamus. This *entry zone* has been variously called Ncl. ventralis caudalis parvocellularis externus (V.c.pc.e, Hassler, 1959), ventral medial nucleus (VM, Gallay et al., 2008) or ventral posterior inferior nucleus (VPI, Jones, 1985). The upper portion of VPI has also been described as relay for vestibular input that projects to the vestibular cortex (Deeke et al., 1974). Forutan and Mai (2012) have referred to the area where cerebellar fibers enter thalamus, as Ncl. ventrolateralis basalis (VLb) in order to emphasize the relationship with the entrance of the adjacent pallidal fibers in the Ncl. ventroanterior basalis (VALb) and the sensory fibers in the Ncl. ventroposterior basalis (VPb).

The ventrolateral region displays rather good concordance W_max_ = 0.71 with strong subdivision realtionship W_asym_ = 0.26.

##### Sensory thalamus

The sensory thalamus represents the main relay of the thalamus for somatosensory and viscerosensory afferents. It is described as *ventroposterior complex* (VP, TNA) because of the multiple well-delimited and characterized nuclei. We include also the special sensory nuclei for vision and audition.

VP is histologically separated into the *lateral ventroposterior nucleus* (ventral posterolateral nucleus, VPL, TNA), the *medial ventroposterior nucleus* (ventral posteromedial nucleus, VPM, TNA) and two smaller parvocellular divisions that were termed the external and the internal divisions of the ventrocaudal nucleus (V.c.pc) equivalent to the *ventral posterior inferior nucleus* (VPI, TNA) and the *medial ventroposterior nucleus, parvocellular part* (*ventral posteromedial nucleus, parvocellular part*, VPMpc, TNA) (Welker, 1973; Kaas et al., 1984; Jones, 2007; see Lenz et al., 2010), respective VPMpc and VLb (Mai and Forutan, 2012). These nuclei provide the receptive area for the spinal, lemniscal and trigeminal fibers.

VPL has been divided into several subdivisions (anterior, posterior, medial, lateral) on the basis of size, density, molecular properties and distribution of cells as well as by their responses to cutaneous stimuli (Jones, 2007). The distinction between the cerebellar territory (VL) and the anterior part of VPL can be made by the transition from the large neurons in VL (Lenz et al., 2010) to the mixed large and small-sized neurons in VPL. Histochemically, there is a difference in the AChE-reaction: low in VLp, very intense in VPL.

VPM receives the ascending secondary trigeminal afferents via the trigeminal lemniscus from the head, face, and intraoral structures.

VPL and VPM are separated by a narrow cell-poor septum (lamella arcuata) that is well seen only during fetal development as distinct (CD15-negative) lamina. Against the centromedian nucleus VPM is delimited by a branch of IL (lamella intermedia, Schnopfhagen, 1877, Figure 6a). Medially and ventrally VPM abuts on VPMpc. Located dorsally is the anterior pulvinar (APul). VPM shows an intense immunoreactivity against parvalbumin but is calbindin-negative (Morel et al., 1997; Münkle et al., 2000).

*Superior ventroposterior nucleus* (VPS). Proprioceptive or kinestetic fibers mediating depth sensitivity project to an area anterior and dorsal to VPM and VPL (at the border with the lateral VL). These fibers are joined by those from the vestibular nuclei. The field where neurons are localized that respond to cutaneous and kinesthetic stimulation can be registered to the superior, anterior or oral part of VP. This part has been termed V.c.e.a, VPS, VPO (oral part) or “shell” region (Hassler, 1959; Jones and Friedman, 1982; Kaas et al., 1984; Jones and Macchi, 1997; Jones, 2007). A precise anatomic delineation of this “deep receptor zone” has not yet performed.

The parvocellular extension of the ventral posteromedial nucleus (VPMpc) is located below CM between the VPM laterally and the subparafascicular nucleus (SPF) medially. It receives general and special visceral afferents and is regarded to serve as thalamic taste area (Pritchard, 2012). From the SPF it is distinguished by its synaptophysin immunoreactivity, whereas SPF is positive for substance P and tachykinin (Mai et al., 1986; Hirai and Jones, 1989).

The spinal, lemniscal and trigeminal afferents to the sensory thalamus are difficult to separate in humans. The different components were therefore lumped together and their portal of entry is described under various names (ventrobasal complex, ventrocaudal complex, posterior nucleus, Burton and Jones, 1976; Basalis complex, Percheron, 2004; ventromedial posterior nucleus, VMpo, Craig et al., 1994; Blomqvist et al., 2000; TNA, 2017). We propose the term basal ventroposterior nucleus (VPb, Mai and Forutan, 2012). For detailed discussion see (Lenz et al., 2010).

The maximal Wallace index for the VP region reads W_max_ = 0.71 and the asymmetry Wallace index is W_asym_ = 0.30. These values indicate strong concordance between the atlases with frequent subdivision relationships between the areas from different atlases.

##### Metathalamus or geniculate region

The term metathalamus denotes two highly differentiated regions related to the lateral thalamus: the lateral and the medial geniculate bodies. The correctness of both terms has been questioned since they may imply that both regions are no ordinary or integral parts of the lateral thalamus (Kuhlenbeck, 1935; Hassler, 1959; Anthoney, 1994). The term “geniculate bodies” is used because they comprise not only the respective nuclei but also derivatives of the dorsal and the ventral thalamus.

The lateral geniculate body (LGB) forms a landmark structure at the ventrolateral and posterior surface of the diencephalon. LGB is composed almost exclusively by the lateral geniculate nucleus (LGN; more precisely the dorsal lateral geniculate nucleus, LGD). The ventral lateral geniculate nucleus (LGV) which is obvious in most mammals is presented in the human brain as pregeniculate nucleus (PG). LGD is triangular in shape and appears in coronal sections as a layered structure that is bent on itself.

The LGD can be divided into six visibly distinct layers (laminae), labeled 1 to 6 from ventral to dorsal. Crossed and uncrossed retinal fibers enter a hilum on its ventromedial surface and terminate in different laminae of the LGN: layers 1, 4, and 6 receive axons from the contralateral eye and layers 2, 3, and 5 receive axons from the ipsilateral eye. The two ventral layers contain relatively large neurons and are termed magnocellular layers (M1, M2). The dorsal layers consist of small cells and are denominated as parvocellular layers (P3 to P6). Intercalated between each magnocellular and the parvocellular layers are the koniocellular layers K1-K6 (Hendry and Reid, 2000).

The *pregeniculate nucleus (PG)* lies as a small and narrow band of cells at the dorsolateral margin of the LGD. It is composed of two parts that were described by Balado and Franke (1937) as loose and dense components, and by Hassler (1959) as Ncl. geniculatus griseus and fibrosus. As a remnant of the rodent ventral lateral geniculate nucleus (LGV) it may possibly also represent the primate equivalent of the intergeniculate leaflet (Lima et al., 2012). The location dorsally to the LGD is the result of the rotation of the LGN during development. It is not part of the (dorsal) thalamus like the LGN but a derivative of the ventral thalamus described in murine brain on a developmental basis as prethalamus (Puelles and Rubenstein, 2003).

**Figure 8:**
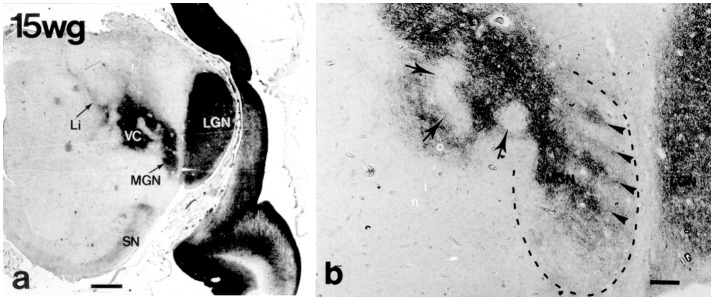
Lateral (LGN) and medial (MGN) geniculate nuclei at 15 weeks of gestation (CD15 immunoreactivity) (from Mai et al., 1999; with permission). **(a)** The continuity of the future MGN with the ventroposterior complex (VC) is apparent. **(b)** Higher magnification shows that the curled, wavy or band like disposition of CD15 immunoreactivity in the ventral parvosellular subnucleus (arrowheads in area surrounded by dashed line) which might correspond to the tonotopically organized fibrodendritic laminae. Arrows indicate regions extending into VC. Scale bars 5 mm in a and 100 μm in **(b)**.

The *medial geniculate body (MGB)* is the last stage of the ascending auditory pathway. It is recognized as a prominence of the ventrolateral surface of the brain medial to the (intergeniculate) pulvinar. It is demarcated against the lateral geniculate body by myelinated fibers which also surround it at the pial surface but the border against the latero-caudal part of the sensory thalamus is indistinct (Figure 8).

The MGB is ovoid-shaped with an intricate internal organization. Its subdivisions have been described mostly by their topographic position, fiber connections and cell morphology (Le Gros Clark, 1933; Winer, 1984). The human MGB is commonly divided into three major divisions which are denoted as parvocellular or principal (lateral) division with major ventral and dorsal components (MGV, MGD) and a magnocellular (medial) division (MGM). Most authors add the suprageniculate-limitans nucleus as the fourth division, the Ncl. geniculatus medialis limitans of Hassler (1959). Alternative terms for the dorsal and ventral divisions of the principal nucleus were the fibrosus and fasciculosus nucleus, respectively (Hassler, 1959).

Cytoarchitectonic analysis results in a much more elaborate organization with additional parcellations especially within the principal division (Morest, 1964; see Harrison and Howe, 1974; Winer, 1984). MGD is very complex with up to 10 subdivisions distinguished (Malmierca and Hackett, 2010). Its main afferents stem from the inferior colliculus; its main target is the auditory association cortex, AII. MGV is the target of fibers of the core ascending, tonotopic information-bearing, auditory pathway from the central nucleus of the inferior colliculus which end within rows of tonotopically organized fibrodendritic laminae. These laminae can be visualized by means of CD15 immunoreactivity (Figure 8). The target of the efferents is the primary auditory cortex, AI.

The representation of the MGB in the atlases shows many variations with respect to parcellation. Of the authors who participated in the analysis of a single brain (Dewulf, 1971) only Hopf and Macchi distinguished subdivisions of MGB. Interestingly, however, were the differing locations of subareas within the MGB complex: whereas Hopf depicted the magnocellular division along the lateral margin next to the LGB, Macchi delineated this division on the medial margin, an area marked by Hopf as limitans division. Hassler (1959); Hassler et al. (1979) and Van Buren and Borke (1972) illustrated the magnocellular division (antero) dorsomedially, adjacent to the ventrocaudal nucleus which contrasts Morel (2007) and Amunts et al. (2012) who depicted this division ventrolaterally along the pial surface of the MGB. Most authors describe the magnocellular division as situated medioventrally (Winer, 1992). The imprecision of the topographic definition of subnuclei is noteworthy in view of the substantial results regarding the development and immunohistochemical properties of the human MGB (Mai et al., 1999; Jones, 2003).

The maximal Wallace index for the metathalamus is W_max_ = 0.84 and the asymmetry Wallace index is W_asym_ = 0.32. These values show the highest concordance between the atlases and strong subdivision relationships between the areas from different atlases, reflecting multiple numbers of subdivisions for LGB and MGB areas.

#### Posterior Region

The *pulvinar nuclei* (Pu) form a large, heterogeneous group of nuclei in the posterior region without clear distinction between subregions. The segmentation of the posterior region is normally based on topographic parameters. TNA distinguishes between the pulvinar (with *medial, lateral, anterior*, and *inferior* nuclei) and the lateral posterior nucleus (LP).

The medial and lateral pulvinar nuclei representing the most extensive nuclei are separated by their fiber density. The anterior pulvinar nucleus corresponds to the Ncl. pulvinaris oralis of Hassler (1959); however, he also designated the corresponding location as Ncl. ventro-caudalis portae (Hassler, 1977; Figure 2). The inferior pulvinar nucleus (Olszewski, 1952; Jones, 1985; Morel et al., 1997) occupies the ventrolateral portion of the pulvinar, positioned close to the brachium of the superior colliculus. The rostral part, intercalated between the medial and lateral geniculate bodies, is described as *intergeniculate* pulvinar.

The human lateral posterior nucleus (TNA, Hirai and Jones, 1989; Morel et al., 1997; Jones, 2007) is regarded as part of the pulvinar and was therefore designated as oral or anterodorsal pulvinar nucleus (Percheron, 1997, 2004; Mai and Forutan, 2012). It corresponds to the Ncl. dorsalis caudalis (Hassler, 1959; Feremutsch and Simma, 1971; Hopf et al., 1971; Van Buren and Borke, 1972). Morel et al. (1997) integrate within the posterior group besides the pulvinar and the lateral posterior nucleus also the posterior complex (Li, Sg, Po) and the geniculate nuclei.

The maximal Wallace index for the P region is W_max_ = 0.80 and the asymmetry Wallace index is W_asym_ = 0.27. These values stand for strong concordance between the atlases and moderately frequent subdivision relationships between the areas from different atlases.

### Concordance Analysis

#### Area (Local)-Level Concordance Analysis

**Figure 9:**
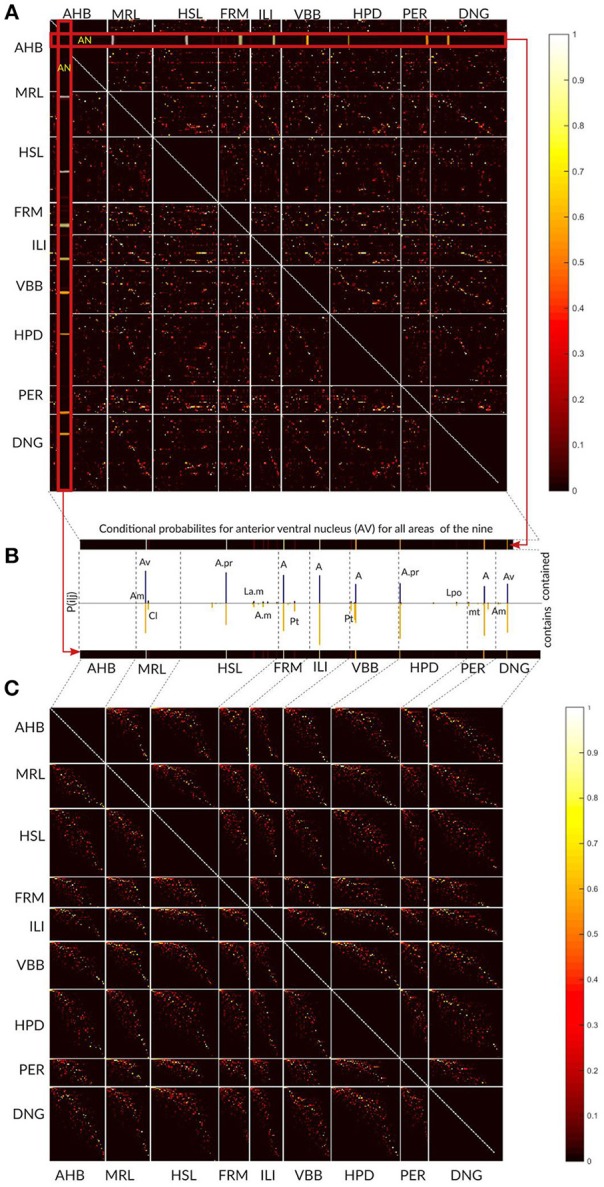
Local area level concordance analysis of the nine atlases shown as image representing the non-symmetric concordance matrix P. **(A)** Each pixel in the image specifies the Pij value by its color. The Pij value expresses the probability of a voxel for being in area ***i*** in one atlas given that it is in area ***j*** in other atlas. Each row and column represent one specific area in an atlas. Areas belonging to an atlas are grouped together and the borders between the atlases are denoted by white lines inducing the appearance of the rectangular blocks in the image. **(B)** Here we show for the anterior ventral nucleus (AV) area from Atlas of the Human Brain how the values in the matrix should be interpreted. The row and column in the matrix that correspond to the AV values (red zoomed rectangles) are displayed as the bars. The color of the pixels in the row and the column (from yellow to red) determines the height of the bars in the plot (see the color bar on the right). The blue bars specify the proportion of AV comprised in other regions, and the yellow bars (below) indicate the proportion of other regions comprised in the AV region. The labels above the bars correspond to the significantly overlapping regions from the other atlases. **(C)** The matrix is shown after modifying the order of the areas independently within each block. The non-zero P_ij_ values form a pixel cloud centered around the diagonal of the block. The brightness of this cloud shows the level of correspondence between the atlases and the width of the cloud approximates the frequency of the subdivision configurations between the atlases.

The overall results of the area-level analysis across the nine thalamic parcellations are depicted in the Figure 9A. The conditional probabilities P_ij_ for all areas are represented as a matrix and visualized as colored image. Each pixel in the image specifies the Pij value by its color. The Pij value estimates local concordance between two areas. It expresses the probability of a voxel for being in area ***i*** in one atlas given that it is in area ***j*** in other atlas. Each row and column represent one specific area in an atlas. Areas belonging to an atlas are grouped together and the borders between the atlases are denoted by white lines inducing the appearance of the rectangular blocks in the image. The number of rows and columns in the image belonging to one atlas reflects the number of areas in this atlas. Colored (non-black) pixels point to areas displaying some degree of spatial overlap. The color variations indicate frequent existence of partial overlap between the areas.

In Figure 9B we used the anteroventral nucleus (AV) of the Atlas of the Human Brain as an example how the entries in the matrix should be interpreted. The row and column in the matrix that corresponds to AV values (red zoomed rectangles) are displayed as the bars. The color of the pixels in the row and the column (from yellow to red) determines the height of the bars in the plot (compare to the colorbar on the right).

The ordering of areas as shown in Figure 9A is rather haphazard, and thus direct visual estimation of the extent of correspondence between two atlases is difficult. Re-arrangement of the rows and columns, i.e., modifying the order of the areas in the atlases, provides a straight-forward interpretation of the image structure as correspondences between the atlases.

We used a singular value decomposition based heuristic from Bohland et al. (2009) to re-order the rows and columns of each rectangular block. This transformation forces the areas with high concordances toward the diagonal and we minimize the overall distance of non-black pixels from the diagonal of that block (Figure 9C).

#### Cluster (Group)-Level Concordance Analysis

To make the concordance analysis more visually tractable we have extended the local area level analysis by the group-level concordance analysis. This analysis was performed separately for 11 groups of regions (see in Material and Methods). The composition of the groups follows the Supplementary Table 1. For each group we determined Wallace maximal index W_max_ and Wallace asymmetry index W_asym_ (Table 2A).

The highest W_max_ values are observed for the MD (0.85), GM/GL (0.84), VAM (0.79), and P (0.80). These values indicate very high concordance, i.e., predictability of the atlases within these regions (Table 2A). We also observe above the chance high W_asym_ values for the following areas: MD (0.35), VAM (0.28), and P (0.26) indicating that many areas in this groups display multiple subset configurations, i.e., an area in one atlas contains multiple areas from another atlas. For example, four atlases divide the area MD into two or more subareas. These subdivisions are responsible for the high W_asym_ value of the M cluster. The asymmetry values for the IL and VAL are below the 95th cut-off threshold indicating that the subdivision configuration in the clusters cannot be distinguished from random thalamus parcellations.

#### Global (Thalamus)-Level Concordance Analysis

The global concordance analysis estimates the inter-atlas correspondences. The results of the global concordance analysis are presented in the Table 2B for W_max_ and the Table 2C for W_asym_. The values in the Tables 2B,C are reported with respect to the chance distribution of random parcellations. Values that exceed 5 percent chance to originate from random parcellations are reported in gray color and in brackets. All inter-atlas concordances W_max_ are above the 5 percent cut-off value for chance distributions of random parcellations. Twelve W_max_ values are not clearly distinct from asymmetries of random parcellations and are reported in gray color.

**Table 2B:**
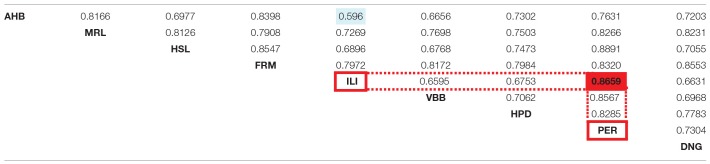
**Global concordance between atlases**.

*Quantification of inter-atlas concordance using the adjusted Wallace index W_max_. The index W_max_ is computed between pairs of whole atlas parcellations and indicates the mean maximal concordance between all pairs of atlas areas. In each cell the values in the upper diagonal entries are the concordance indices for particular pairs of the atlases. The maximal W_max_ value (red) is observed between PER and ILI atlases. The minimal W_max_ is observed between AHB and ILI atlases (blue). For the atlas labels on the diagonal see Table 1*.

**Table 2C:**
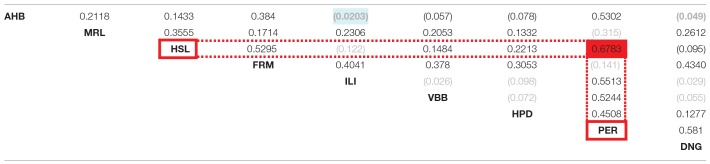
**Global asymmetry of concordance between pairs of atlases**.

*Each cell of the table displays the asymmetry Wallace index W_asym_ for the selected pair of the atlases. The index W_asym_ is computed between pairs of whole atlas parcellations and indicates the mean maximal asymmetry between all pairs of atlas areas. High values of W_asym_ reveal frequent subdivision configuration between atlaes. The maximal W_asym_ value (red) is observed between PER and HSL atlases. The minimal W_asym_ is between AHB and ILI atlases (blue). The values below chance threshold (the 95th percentile of the chance distribution of random parcellations) are in brakets and colored gray*.

The high number of values makes it difficult to see any characteristic pattern between the atlases. To facilitate the detection of such characteristic patterns we use multi-dimensional scaling (MDS). The MDS transforms the concordance values W_max_ and W_asym_ between the atlases into positions of a 2D space such that more similar atlases occupy nearby points in this two-dimensional space while less similar atlases become more distant. In this approach the atlas similarities expressed as closeness of W_max_ and W_asym_ values are translated into nearby positions in the 2D space.

**Figure 10:**
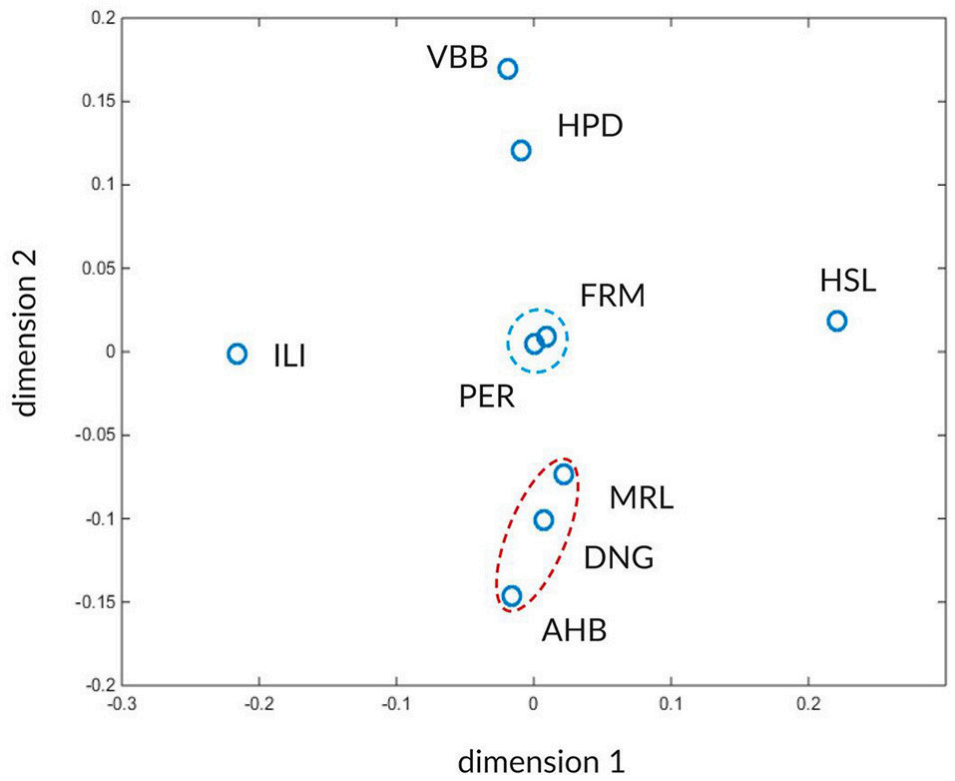
Visualization of inter-atlas relationships using multi-dimensional scaling. The atlases are shown in a 2-D landscape computed from distances derived from the W_max_ and W_asym_ values (Tables 2B,c). Atlases that can mutually be better predicted from each other and share similar asymmetry values reside closer in this space. The two recognizable clusters are indicated by red and blue dashed ellipsoids. The arbitrary dimensions one and two give coordinates for the projected atlas similarity values in the 2D space.

The results after applying MDS using the global concordances W_max_ and asymmetries W_asym_ from the Tables 2B,C are shown in Figure 10. Two distinct clusters of atlases are marked by red and blue dashed ellipses, surrounded by dissimilar atlases. The red cluster contains the FRM and PER atlases and the blue cluster includes AHB, DNG, and MRL atlases.

**Figure 11:**
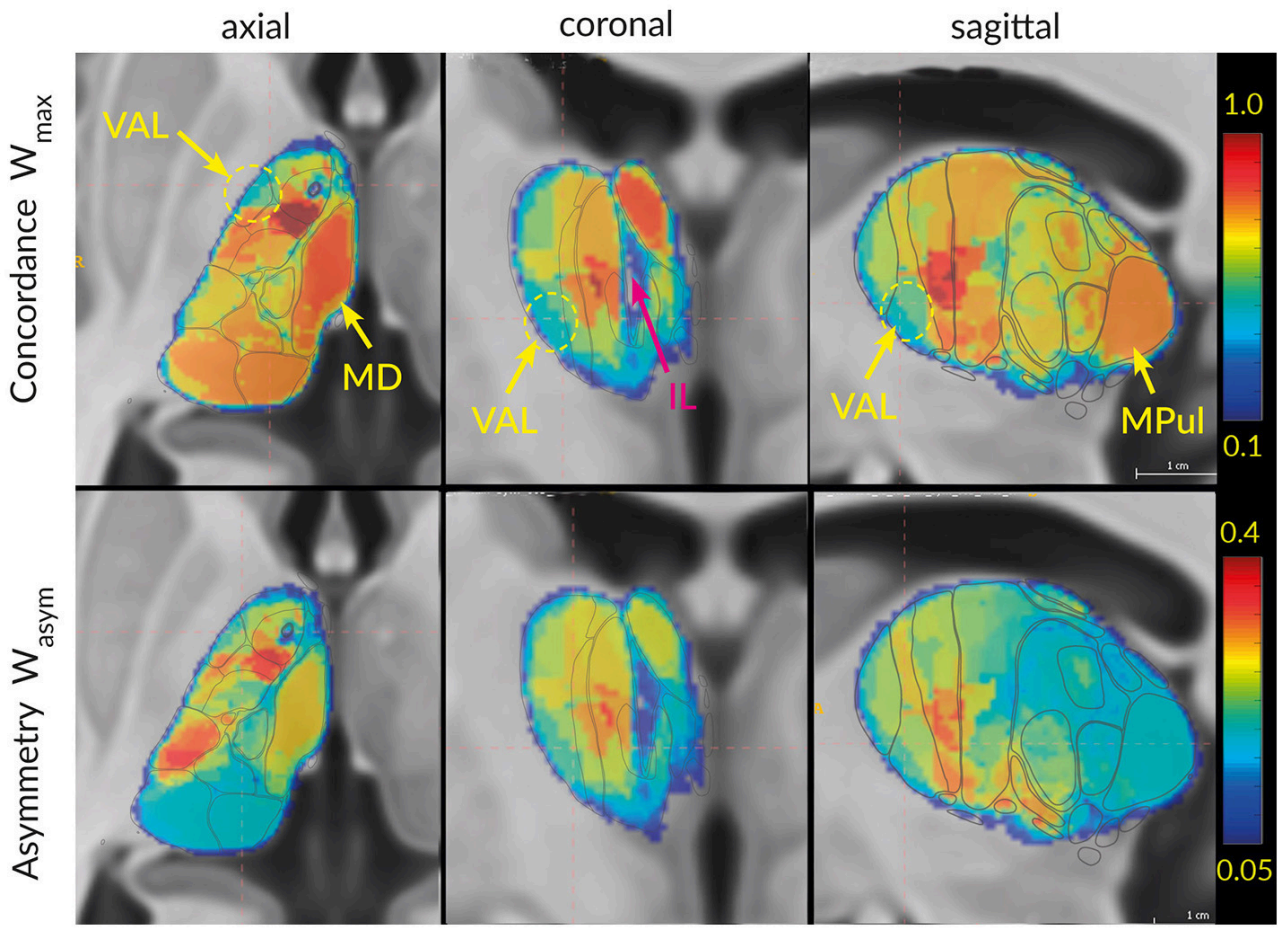
Visualization of W_max_ (top row) and W_asym_ (bottom row) in 3D space. The regions with high concordances and asymmetry (MD, AV) can directly be identified in the slices as areas with strong red color. The ellipsoid and arrows point to the region with the lowest concordances: lateral ventroanterior nucleus (VAL) and intralaminar formation (IL).

In addition to the inter-atlas predictability from Tables 2B,c and to the two clusters discriminated by MDS we are interested in the spatial distribution of the atlas concordances across the thalamus volume. To this end we computed the average global atlas concordance and asymmetry for each voxel of the thalamus. The spatial distributions of W_max_ and W_asym_ exemplify the results of the cluster concordance analysis (Figure 11). Clusters of high (M, VAM) and low (VAL) concordances are easily distinguishable in the three planes. Similarly the asymmetry values mirror the results of the cluster concordance analysis. For example the high W_max_ and W_asym_ values of the M cluster follow the expected borders of the MD region. What the cluster concordance analysis does not show are the remarkable gradients in the concordance and asymmetry distributions within the clusters. Particularly the strong concordance and asymmetry focus in the VAM subregion dominates the figure.

## Discussion

### Consistencies and Differences of Thalamus Delineations May be Resolved by Multi-Layered Nomenclature Definition

The description of the thalamus in humans rested in the past on the analysis of individual brains. Our representation of the maps published by various authorities illustrates their enormous discrepancies. These arise in part from the inherent variability of the object but are above all the result of personal interpretation of the findings. As a consequence, the wording used for the description of thalamic features is inconsistent and, in addition, compromised by historic trends influenced by different “schools” (Anthoney, 1994). Same terms may have well-accepted but conflicting meanings and similar features may have diverse interpretations. This makes the correlation and thus the topographic comparison of the results from different research difficult and the application of the diverse tables of synonyms questionable. The differing understanding of the topographic organization of the human thalamus and the dissenting terminological concepts makes it highly unlikely that a harmonized and generally accepted agreement is achieved by renewed discussion of disagreements regarding the meaning or appropriate usage of terms.

Nevertheless, all studies reside on thorough analysis and comprehensible concepts. It is therefore demanded to look for ways to discuss terms and the labeled structure together. To arrive at a more consistent interpretation of histological and radiological features and of their topographic definition a new approach is suggested. We argue that the greatest hindrance for a commonly accepted interpretation of the human thalamus, the variation of neighborhood relations in the individual space, can be overcome by their registration into a common space.

For our approach we exploited the delineations of thalamic nuclei from serial sections that were published from well-reputed researchers. The three-dimensional reconstructions from their maps were registered into a standard ICBM/MNI152_2009b space because it is openly accessible and has been used for the registration of our individual “Atlas of the Human Brain” (AHB, Mai et al., 2016) and our average atlas of the human brain (Mai and Majtanik, 2017).

The registration of the different atlases into the same space allowed us to evaluate consistencies and differences of delineations and neighborhood relations in the same (standard) frame but in relation to the original published materials. It is a matter of course that the three-dimensional reconstruction of this heterogeneous material introduces problems regarding the accuracy and consistency of our result. The registration of the atlases in the standard space does not mean the perfect match of the spatial relationship between the atlases.

Given this limitation we were able to estimate the relative spatial overlaps between the nine atlases within the standard MRI volume. We parcellated this volume into “clusters” (Supplementary Table 1) that were developed on the basis of the interpretation of correlating terms, i.e., territories. This process renders susceptibility to personal bias effects. This effect must also be accounted for because the comparison between the atlases–as described here–is in relationship to the AHB or, in the case of the lateral region, to the delineation provided by Hassler (Figure 4).

Based on this material we have performed systematic quantitative analysis of the relationships between different anatomical atlases of the thalamus. We claim that not the disagreements in terminology are the major cause of the brain atlas concordance problem but the definition of underlying partition volumes of the brain anatomy (e.g., atlases).

A possible solution of this concordance problem is a multi-layered nomenclature definition. For regions of high concordance only one nomenclature base layer is required. This base nomenclature layer may possibly be generated by some concordance optimization algorithms and be further curated by experts. For the regions of low concordance multiple nomenclature layers should be created to account for the heterogeneity of defining partition concepts.

We aimed to follow the recommendations of the revised terminology in the Terminologia Anatomica (TNA, 2017) made by the Working Group Neuroanatomy of the Federative International Programme for Anatomical Terminology (FIPAT) of the International Federation of Associations of Anatomists (IFAA). As consquence of the screening of the final scheme of nine atlases and the results from the quantitative computational analysis we amended some of those recommendation. In Supplementary Table 1 we have summarized the selected terms for the subdivisions of the human thalamus and have included references which show the emergence of the listed terms together with some equivalent designations. We understand this recommendation as a base layer terminology that has to be extended by additional layers.

### Concordance Analysis

*Area* (local)-level analysis evaluated pairwise spatial overlap between two areas by conditional probabilities and revealed that the one-to-one relationship between the areas is rarely observed in the data. The voxels within an area in one atlas mostly map to multiple areas of another atlas (Figure 9). To visualize this relationship we developed a meaningful visualization of the multiple area mapping by re-ordering columns and rows of blocks in matrix P (Figure 9C). For each block we can observe an elongated diagonal cloud of nonzero voxels. The brightness of the cloud indicates the level of correspondence between the areas and the width of the cloud indicates the frequency of the subdivision configurations between the atlases. Bright diagonal clouds indicate high correspondence and high W_max_ values. Broad diagonal clouds hint to frequent one-to-multiple subdivision configurations and is reflected in high W_asym_ values.

The *cluster* (group)-level analysis focuses on the correspondences between multiple areas belonging to a region with common anatomical characteristics. The concordance analysis of such clusters imposes a spatially constrained view on the parcellation equivalence and stability between various atlases. To explore the cluster correspondences we developed the W_max_ and W_asym_ indices. The W_max_ and W_asym_ capture similarity between several areas while allowing for area refinement in one atlas relative to another. The W_max_ captures similar correspondence properties as the S-index defined by Bohland et al. (2009) and does not penalize the subdivision configurations in clusters. Our W_asym_ index directly quantifies the one-to-many subdivisions between the areas in a cluster. A cluster with low concordance values W_max_ points to the fact, that the underlying characteristics of the constituent areas are highly variable between the atlases. A large W_asym_ value for a cluster indicates very strong one-to-many relationships between areas from different atlases. For example, four atlases divide the area MD into two or more subareas. These subdivisions are responsible for the high W_asym_ value of the MD group. The lowest W_max_ values are displayed by the IL (0.55) and VAL (0.61) group (Table 2A and Figure 11). The low predictability of the IL and VAL cluster points to highly heterogeneous concepts for these areas in the nine atlases.

For direct comparison of the different cluster parcellations we extended the cluster analysis with detailed estimation of the conditional probabilities between the clusters of the “Atlas of the Human Brain” and clusters of the other atlases. Supplementary Table 3 lists the conditional probabilities and corresponding areas for all eleven groups. We observed that clusters with high W_max_ values in Table 2A also show high conditional probabilities in Supplementary Table 3. The high W_asym_ values of the clusters in the Table 2C are reflected by the increased numbers of overlapping areas in the Supplementary Table 3.

The *global* (thalamus)-level concordance analysis provided quantitative estimates of the inter-atlas correspondences and asymmetries. The large number of pair-wise atlas comparisons shown in Table 2B for W_max_ and Table 2C for W_asym_ makes it difficult to directly identify more complex patterns of equivalences. To facilitate the recognition of complex correspondence patterns we mapped the atlases into a two-dimensional space using multi-dimensional scaling (Figure 10), such that atlases with more similar regions (more overlapping, less asymmetric) appear in closer proximity with one another compared to atlases with less overlapping regions. This intuitive graphical representation allows to capture similarity in concordance and asymmetry between atlases. The MDS revealed two clear clusters (Figure 10). The blue cluster contains the FRM and PER atlases and the red cluster includes AHB, DNG, and MRL atlases. The characteristic pattern for the blue cluster originates in very high W_max_ and W_asym_ values of the FRM and PER atlases as compared with the other atlases except for single low asymmetry value of W_asym_ = 0.14 between the FRM and PER atlases. The characteristic pattern for the red cluster derives from high values of W_max_ and low values W_asym_ within of the cluster combined with very high values of W_asym_ to the atlases of the red cluster. Atlases outside of the two clusters do not consistently show these patterns.

We interpret these two characteristic patterns in the following way: The atlases combined within the red cluster contain high and those within the blue cluster contain low number of areas. Hence the areas of the red cluster are predominantly small and those within the blue cluster are large. High W_max_ values indicate good concordance between the atlases within of the clusters with low frequency of subdivision configurations. In contrast, the high W_asym_ values between the clusters indicate multiple subdivisions of the blue cluster atlases by the red cluster atlases.

We can also derive the high W_max_ and the low W_asym_ properties of the clusters from the Figure 4. The extension, the configuration and the edge orientation of the areas of the blue MDS cluster atlases (FRM, PER) show broad agreement (Figures 4B,D, W_max_ = 0.83). The number of delineations in the cross-sections is more similar between FRM (32 areas) and PER (29 areas) atlases compared to the other atlases (more than 42 areas). Analogous observation holds for the red MDS cluster (AHB, MRL, DNG) in Figures 4E,G,H. In this cluster the number of areas is two times the number of areas of the blue cluster. The overall configuration of the colored regions does not display extreme differences between the three atlases. Noteworthy is the high similarity of the colored region configurations and extensions between the HSL atlas and the blue MDS cluster. The concordance W_max_ = 0.89 between the HSL and the PER atlases is the highest observed W_max_ value in the Table 2B. The W_max_ of 0.85 between HSL and FRM support this observation. Three times more areas of the HSL atlas cause strong subdivision configurations with the blue cluster and result in high W_asym_ = 0.67 for the PER atlas and W_asym_ = 0.53 for the FRM atlas. This dissimilarity places the HSL atlas outside of the blue MDS cluster.

### Consequences of the Concordance Analysis Approach for the Terminology of the Thalamus

Our classification of substructures into clusters reflects the compromise between the diverging views between our own interpretation and preceding delineations and terms, which often changed over the years. The results of our concordance analysis provide values that request renewed and focused analysis of the parcellation of the human thalamus. The areas with low concordance values W_max_ derive from non-equivalent parcellation concepts resulting in questionable or even diverging labeling. To come closer to unequivocal definitions of the thalamic nomenclature we have to focus on these low concordant areas and must analyze the underlying parcellation concepts to select the most appropriate one with its corresponding name. We suggest including additional parameters which support the registration process. As pointed out, the intralaminar formation presents a key for the parcellation of the human thalamus. The formation is in part identified in the ICBM/MNI152_2009b template. Distance measures to other discriminated structures could constrain the registration process and provide improved inter-space probability.

Provided that the relative position of delineated areas or points of interest remain preserved after the registration process and that their topography matches with the reference cases we can claim first, the neglect or even absence of an individualized anatomy and of anatomic variables and second, the possibility to statistically estimate the degree of concordance between structures.

As final goal of our approach we emphasize that the spatial extension of an area becomes the prime representative of underlying defining concepts and that the anatomical labels forfeit their dominance.

**Figure 12:**
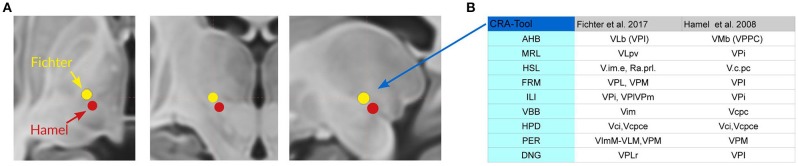
Active deep brain stimulation (DBS) sites in tremor patients from two studies (Hamel et al., 2007; Fiechter et al., 2017) attributed to the VIM region. **(A)** Positions of the DBS stimulation sites in the MNI space. The coordinates of the DBS target in MNI space for Fiechter et al. (yellow) x = 14.3 mm, y = −17.40 mm, z = −2.17 mm and for Hammel et al. (red) y = 12.7 mm, y = −19.6 mm, z = −4.38 mm. **(B)** Anatomical characterization of the targets (Table 2D) presented in an analysis tool. Such anatomical characterization is available for each voxel in thalamus.

Today there is increased need to translate anatomical information from classical neuroanatomical fields to new use cases. The use of single names to characterize regions of interest is problematic and may lead to obvious discrepancies between anatomical nomenclature reference and anatomical characterization of areas. As example we have analyzed the topography of the so-called “VIM area.” This area is defined in correspondence to the area mapped in the atlas from Hassler (1959, 1977). It is often selected as target for deep brain stimulation (DBS) in cases of movement disorders. The outcome of the stimulation is highly correlated with the precision of the electrode targeting. The clinically “effective” area does, however, seldom match the area defined in the anatomical atlas of Hassler. Analysis of the “VIM” target coordinates with best therapeutic effects render coordinates mostly outside the original definition (Figure 12). The effective target suggested by Fiechter et al. (2017) coincides with VIM only in three out of nine atlases. This example illustrates, that characterization based on traditionally ascribed names, must not reflect the real position in the standard space. The difference between the reported label (VIM) and real position is space is striking (Table 2D).

**Table 2D:** Anatomical characterization of the stimulation sites for tremor patients in the studies of Fiechter et al. (2017) and Hamel et al. (2007).

	**AHB**	**MRL**	**HSL**	**FRM**	**ILI**	**VBB**	**HPD**	**PER**	**DNG**
Fiechter et al., 2017	VLb (VPI)	VLpv	V.im.e, Ra.prl.	VPL, VPM	VPi, VPlVPm	Vim	Vci,Vcpce	VImM-VLM, VPM	VPLr
Hamel et al., 2007	VMb (VPPC)	VPi	V.c.pc	VPI	VPi	Vcpc	Vcpci, Zic.Rpl	VPM	VPI

*The labels for the nine atlases are provided in the Supplementary Table 5. The coordinates of the VIM targets in MNI space for Fiechter et al.: x = 14.3 mm, y = −17.40 mm, z = −2.17 mm and Hamel et al.: x = 12.7 mm, y = −19.6 mm, z = −4.38 mm*.

With our approach we do not assign “true” labels to the stimulation site but an array of labels along with corresponding concordance for these areas. Such arrays can better inform the researcher about the structure of concept equivalence and facilitates more appropriate interpretation of data and results. Further, the concept of the stimulation site can be extended by aggregation of efficiency outcomes into probabilistic spaces.

This example illustrates the possibility to improve the criteria for the definition of thalamic volumes or subareas by non-morphologic descriptors, e.g., aspects of molecular, connectional or functional organization respecting maximum probability feature maps.

Horn et al. (2017) proposed a tool for characterization of the spatial location of DBS targets by various MNI atlases based on histology, functional or diffusion-weighted MRI and connectome data (Behrens et al., 2003). An estimation of the extent of correspondence with the different atlases may improve the targeting process. We think that a concordance-based measure will provide indicators to reliably assess the quality of specification of areas of interest.

The concordance analysis framework may be extended to the whole brain profiting from developments in the neuroimaging field. The region discriminating concepts, as we already developed for reporting of different views of cortex partitioning (Mai et al., 2016), will be not specific for an area, but defined as multidimensional feature fields within the brain thereby allowing analysis and discrimination of areas by automatic detection algorithms (Glasser et al., 2016). Using such tools spatially defined multi-modal-atlases may be developed that allow mapping of regions defined by a multitude of protocols.

## Author Contributions

All authors listed have made a substantial, direct and intellectual contribution to the work, and approved it for publication.

### Conflict of Interest Statement

JM is CEO of MRX-Brain GmbH, MM is data analyst and AI developer for MRX-Brain GmbH.
