# Supplementary material for: Toward a Common Terminology for the Thalamus
Source: Front Neuroanat. 2019 Jan 11;12:114. doi: 10.3389/fnana.2018.00114 (PMC6336698; doi:10.3389/fnana.2018.00114)
Supplement: Supplementary file 1 [file Table_1.pdf]

Supplementary Material

Towards a Common Terminology for the Thalamus

Jürgen K. Mai*, Milan Majtanik

*** Correspondence:** Juergen K. Mai: mai@uni-duesseldorf.de

Supplementary Tables

Table 1. Recommended nomenclature for the human thalamic nuclei. Listed in parenthesis are references which show the emergence of the itemized terms together with some equivalent designations.

Thalamus *(His, 1893)*

Intralaminar Formation (IL) *(Sheps, 1945, H: medial nuclear group;*

ILA anterior group of the intralaminar nuclei (TNA)

- CeM central medial nucleus *(Gurdjian, 1927, rt: Ncl. centralis; Rioch, 1929, dg, ct; Le Gros Clark, 1932; Sheps, 1945, H)*
- CeL central lateral nucleus *(Rioch, 1929, dg, ct; Le Gros Clark, 1932; Sheps, 1945, H; Friedemann, 1911, mk: Ncl. paralamellaris)*
- PCe paracentral nucleus *(Gurdjian, 1927, rt; Rioch, 1929, dg, ct; Sheps, 1945, H)*
- Cuc cucullar nucleus *(Hassler, 1959)*
- Fa fasciculosus nucleus *(C. Vogt, 1909, mk; Le Gros Clark, 1932: Ncl. ventralis pars anteromedialis)*

ILCe central group of intralaminar nuclei (TNA)

- CM centromedian nucleus *(Luys, 1865, centre médian)*
- PF parafascicular nucleus *(Vogt and Vogt, 1902, H, campus PF; Friedemann, 1911, mk; Sheps, 1945, H)*
- SPf subparafascicular nucleus *(Papez and Aronson, 1934, mk; Rioch, 1929, dg, ct)*

ILP posterior group of intralaminar nuclei (TNA)

- Lim limitans nucleus *(Friedemann, 1911, mk; Rioch, 1929, dg, ct; Sheps, 1945, H)*
- SGe suprageniculate nucleus *(Münzer and Wiener, 1902, rb; Friedemann, 1911, mk; Sheps, 1945, H)*

Periventricular Formation (PeF) and Midline Nuclei (MN) *(Nissl, 1889, rb: Mittellinienkerne; Sheps, 1945, H: midline group; Dewulf, 1971, H)*

subependymal nuclei (periventricular gray matter of thalamus, *Sheps, 1945, H*)

- PeV periventricular nucleus *(Sheps, 1945, H)*

dorsal component of midline nuclei

- PT paratenial nucleus (TNA); *(Vogt, 1909, mk; Friedemann, 1911, mk; Gurdjian, 1927, rt; Sheps, 1945, H)*
- PV paraventricular nucleus (TNA); *(Gurdjian, 1927, rt; Sheps, 1945, H; Hassler, 1959, H: Ncl. paramedianus)*

ventral component of midline nuclei

- Re reuniens nucleus *(Malone, 1910, H; Gurdjian, 1927, rt; Grünthal, 1943, H: Ncl. medialis posterior reuniens; Sheps, 1945, H; Hassler, 1959, H: Ncl. endymalis)*
- SM submedius nucleus *(Vogt, 1909, mk; Friedemann, 1912, mk: Ncl. submedialis, mv; Krieg, 1944, rt; Sheps, 1945, H);* medial ventral nucleus *(Gurdjian, 1927, rt; Grünthal, 1934, H)*

Anterodorsal Region (A) *(Friedemann, 1911, mk, pars anterodorsalis; Percheron, 2004, superior region)* (anterior group nuclei, AN; anterior nuclear group, Ncll. anterior thalami) *(Luys 1865: centre antérieur; Burdach, 1822: oberer Kern; Vogt, 1909, mk: partie antéro-dorsale; Crouch, 1934, mk; Walker, 1938, mk: Ncl. lateralis dorsalis)*

- AV anteroventral nucleus *(Gurdjian, 1927, rt; Sheps, 1945, H);* anteroprincipal nucleus *(von Monakow, 1895, ct,dg, H: ant a; Vogt, 1909, mk; Hassler, 1959);* Ncl. dorsalis magnus *(Niimi, 1949).*
- AM anteromedial nucleus *(Sheps, 1945, H;)*
- AD anterodorsal nucleus *(von Monakow, 1895, ct,dg, H: lat.; Gurdjian, 1927, rt; Sheps, 1945, H;)*

DSf dorsal superficial nucleus (*Vogt & Vogt, 1941, H: superficiale caudale; Hassler, 1959);* lateral dorsal nucleus *(Sheps, 1945, H; von Monakow, 1895: noyau antérieure accessoire, ant c; Koelliker, 1896: noyau intermédiaire)*

Medial Region (M) *(Vicq d'Azyr, 1796; Burdach, 1822, Ncl.cinereus internus; Luys, 1890, centre moyen; Foix and Nicolesco, 1925;Grünthal, 1934, H: Ncl. internus)*

MD mediodorsal nucleus (TNA); *(von Monakow, 1895, ct,dg, H: med.a; Vogt, 09; Gurdjian, 1927, rt: Ncl. medialis dorsalis; Vogt & Vogt, 1941, H: mediale dorsale)*

MDM medial mediodorsal nucleus (TNA; magnocellular MD; Ncl. medialis fibrosus, *C. Vogt, 1909, mk*)

MDCe central mediodorsal nucleus (parvicellular MD, Ncl. medialis fasciculosus)

MDL lateral mediodorsal nucleus (TNA; paralaminar [paralamellar] MD; Ncl. densocellularis / multiformis

Lateral Region (L) *(Burdach, 1819-1926, H: äußerer Kern, Ncl. cinereus externus; von Monakow, 1895, H: lateraler Kern; Déjérine & Déjérine- Klumpke, 1895: noyau externe; Vogt and Vogt, 1941, H: laterale)*

Motor Thalamus

VA ventroanterior nucleus (ventral anterior nucleus, TNA; Ncl. ventralis anterior) [indirect striatal and amygdaloid territory] *(Rioch, 1929, dg, ct; Walker, 1938, mk; von Monakow, 1895, H: lateralis b; Grünthal, 1934, H: Ncl. lateralis anterior B; Sheps, 1945, H: Ncl. ventralis anterior;)*

VAM medial ventroanterior nucleus (VAmc, magnocellular ventroanterior nucleus, TNA) (nigral territory) (*Van Buren & Borke, 1972, H: lateropolaris – fasciculosus complex)*

VAL lateral ventroanterior nucleus (VApr, principal ventroanterior nucleus, TNA) (pallidal territory) *(Walker, 1938, mk; Van Buren & Borke, 1972, H: Ncl. ventro-oralis)*

VAb basal ventroanterior nucleus *(Mai & Forutan, 2012).* (VAMb, entry zone of nigral and amygdaloid afferents; VALb, entry zone of pallidal afferents)

VL ventrolateral nucleus (ventral lateral complex, TNA; Ncl. ventralis lateralis) [cerebellar and vestibular territory] *(von Monakow, 1895, H: vent c; Vogt C, 1909, mk: noyau ventral intermédiaire; Walker, 1938, mk; Vogt and Vogt, 1941, H: Ncl. ventrale intermedium; Ncl. ventralis intermedius)*

VLA anterior ventrolateral nucleus (TNA, medial oral division, VLM)

VLP posterior ventrolateral nucleus (TNA, lateral caudal division, VML)

VLPi posterior ventrolateral nucleus, internal part

VLPe posterior ventrolateral nucleus, external part

VLb basal (inferior) ventrolateral nucleus (entry zone of cerebellar fibers) *(Mai & Forutan, 2012)*

Sensory Thalamus

VP ventroposterior nucleus or complex, TNA (ventral posterior nucleus or complex; Ncl. ventralis posterior) [somato- and visceral sensory territory] *(Walker, 1938, mk: Ncl. ventralis posterior; Vogt and Vogt, 1941, H: Ncl. ventrale caudale),* ventrobasal complex *(Rose, 1935, rb: ventrobasal complex [VPL+VPM])*

VPL lateral ventroposterior nucleus (ventralis posterolateral nucleus; TNA; Ncl. ventralis postero-lateralis) *(Foix and Nicolesco, 1925, H; Sheps, 1945, H)*

VPM medial ventroposterior nucleus (ventral posteromedial nucleus, TNA; Ncl. ventralis postero-medialis) *(Sheps, 1945, H) (von Monakow, 1895: vent b, H; von Tschisch, 1896: schalenförmiger Kern; Flechsig,* 1876*: Ncl. semilunaris, H; Koelliker, 1896: Ncl. arcuatus, H; Ncl. posterointernus)*

VPS superior ventroposterior nucleus *(Sheps, 1945, H: ncl.ventralis posterior intermedialis; Kaas et al., 1984, mk; Kaas, 2012, H) (Friedman & Jones, 1981, mk: VP "shell"; Dykes, 1983, oral ventroposterior nucleus, VPO)*

VPMpc medial ventroposterior nucleus, parvocellular part (ventral posteromedial nucleus, parvocellular part, TNA; Ncl. ventroposterior medialis, pars parvocellularis *(Friedemann, 1911, mk; Olszewski , 1952, mk),* Ncl. ventralis arcuatus *(Aronson and Papez, 1934);* Ncl. ventromedialis posterior *(Rose, 1935, rb),* ventral medial basal nucleus, VMb *(Jones, 2007; Berman & Jones, 1982, ct: basal ventromedial nucleus) (Toncray and Krieg, 1946, H: Ncl. semilunaris accessorius)*

VPb basal ventroposterior nucleus [VMpo] (entry zone of sensory afferents) *(Mai and Forutan, 2012)*

Metathalamus (Geniculate Region)

LG lateral geniculate nucleus *(Gratiolet, 1839: corps genouillé externe; Henle, 1871: Corpus geniculatum laterale; Meynert, 1870, H: Corpus geniculatum externum)*

LGD dorsal lateral geniculate nucleus, TNA *(Longet, 1847-1849; Luys, 1865; Meynert, 1870, H: Corpus geniculatum internum; Henle, 1871: Corpus geniculatum laterale)*

magnocellular layers (*von Monakow, 1885, ct,dg, H; Malone, 1910)*

parvocellular layers *von Monakow, 1885, ct,dg, H; Malone, 1910)*

koniocellular layers *Hendry and Reid, 2000*

PG pregeniculate nucleus *(Vogt and Vogt, 1902, H: Griseum praegeniculatum)* (LGV, ventral lateral geniculate nucleus)

MG medial geniculate nucleus, TNA *(Malone, 1910)*

MGD MG, dorsal division *(Vogt, 1909, mk: Noyau fibreux)*

MGV MG, ventral division *(Vogt, 1909, mk: Noyau fibreux)*

MGmc MG, magnocellular division *(Vogt, 1909, mk: Noyau gris)*

MGL MG, limitans division *(Hassler, 1959, H)*

Posterior Region (P) *(Luys, 1865, centre postérieur)*

Pul pulvinar nuclei, TNA (pulvinar *(Burdach, 1822, H; Grünthal, 1934, H)*, pulvinar-LP complex)

APul anterior (oral) pulvinar nucleus, TNA (*Vogt & Vogt, 1941, H)*

ADPul anterodorsal pulvinar nucleus (lateral posterior nucleus) *(Mai and Forutan, 2012, H)*

IGPul intergeniculate pulvinar nucleus *(Malone 1910, H)*

IPul inferior pulvinar nucleus, TNA *(Sheps, 1945, H)*

LPul lateral pulvinar nucleus, TNA (*Sheps, 1945, H)*

MPul medial pulvinar nucleus, TNA *(Sheps, 1945, H)*

SFPul superficial pulvinar nucleus (*Hassler, 1959, H)*

Prethalamus

PRt prereticulate nucleus *(Mai and Forutan, 2012)*

Rt reticulate nucleus *(Arnold, 1851: Stratum reticulatum)*

Fa fasciculosus nucleus *(Hassler, 1959)*

Epithalamus

HB habenular nucleus *(Meynert, 1870: ganglion habenulae)*

HBL lateral habenular nucleus

HBM medial habenular nucleus

Abbreviations: ct: cat; dg: dog; H: human; mk: monkey; rb: rabbit; sh: sheep

Literature cited

Arnold F (1851) Anatomie des Menschen. Bd 2,2

Berman AL, Jones EG (1982) The thalamus and basal telencephalon of the cat. A cytoarchitectonic atlas with stereotaxic coordinates. University of Wisconsin Press, Madison

Burdach KF (1822) Vom Baue und Leben des Gehirns. Bd 2, Dyk'sche Buchhandlung, Leipzig

Crouch RL (1934) The nuclear configuration of the hypothalamus and subthalamus of Macacus rhesus. J. comp. Neurol. 59: 431–449

Dejerine J, Dejerine-Klumpke A (1895—1901) Anatomie des Centres Nerveux. Vol. 1,2. Masson, Paris https://gallica.bnf.fr/ark:/12148/bpt6k76498h/f13.image
Dewulf A (1971) Anatomy of the Normal Human Thalamus. Elsevier, Amsterdam
Dykes RW (1983) Parallel processing of somatosensory information: a theory. Brain Res. Rev. 6, 47–115
Flechsig PE (1876) Die Leitungsbahnen im Gehirn und Rückenmark des Menschen auf Grund entwicklungsgeschichtlicher Untersuchungen. Leipzig, Engelmann
Foix C, Nicolesco J (1925) Anatomie cérébrale. Les noyaux gris centraux et la région mésencéphalo-sous-optique. Masson et Cie, Paris pp. 508–538
Friedemann M (1911) Die Cytoarchitektonik des Zwischenhirns der Cercopitheken mit besonderer Berücksichtigung des Thalamus opticus. J Psychol Neurol (Leipzig)18: 309-379
Friedman DP, Jones EG (1981) Thalamic input to area 3a and 2 in monkeys. J. Neurophysiol. 45: 59-85 PMID: 7205345; DOI: 10.1152/jn.1981.45.1.59
Gratiolet LP (1857) Anatomie comparée du système nerveux considéré dans ses rapport avec l'intelligence , eds Leuret F, Gratiolet LP. Vol 2 (Vol 1, 1839) Ballière, Paris
Grünthal E (1934) Der Zellbau im Thalamus der Säuger und des Menschen. Eine beschreibend und vergleichend anatomische Untersuchung. J Psychol Neurol (Leipzig) 46:41–112
Gurdjian ES (1927) The diencephalon of the albino rat. J Comp Physiol Psychol 43: 1-144
Hassler R (1959) Anatomy of the Thalamus. In: Schaltenbrand G, Bailey P (eds) Introduction to Stereotaxic Operations with an Atlas of the Human Brain. Thieme Stuttgart, pp 230-290
Hendry SH, Reid RC (2000) The koniocellular pathway in primate vision. Annu Rev Neurosci. 23:127-153
Henle J (1871) Handbuch der systematischen Anatomie des Menschen: Nervenlehre, Vol 3, Part 2. Vieweg, Braunschweig (Permalink: http://mdz-nbn-resolving.de/urn:nbn:de:bvb:12-bsb11156731-0)
His W (1892) Zur allgemeinen Morphologie des Gehirns. Arch. f. Anat. u. Physiol. Anat. Abth.
Jones EG (1985) The Thalamus. Plenum Press, New York
Jones EG (2007) The Thalamus. Cambridge Univ. Press, Oxford
Kaas JH, Nelson RJ, Sur M, Dykes RW, Merzenich MM (1984) The somatotopic organization of the ventroposterior thalamus of the squirrel monkey, Saimiri sciureus. J. Comp. Neurol.**226,** 111–140
Kaas JH, and Pons TP (1988) The somatosensory system of primates. Comp. Primate Biol. 4, 421–468
Koelliker A (1896) Handbuch der Gewebelehre des Menschen 2. 6., umgearb. Aufl. Nervensystem des Menschen und der Thiere, Engelmann, Leipzig
Krieg WJS (1944) The medial region of the thalamus of the albino rat. J. comp. Neurol., 80: 381-415 https://doi.org/10.1002/cne.900800307
Le Gros Clark WE (1932) The structure and connections of the thalamus. Brain 55:406-470
Leuret F, Gratiolet LP (1839) Anatomie comparée du système nerveux considéré dans ses rapports avec l'intelligenc, vol 1 Ballière, Paris
Longet F-A (1847-1849) Anatomie und Physiologie des Nervensystems des Menschens und der Wirbelthiere, mit pathologen Beobachtungen und mit Versuchen an Höheren Thieren ausgestattet. Brockhaus & Avenarius, Leipzig
Luys J (1865) Recherches sur le système nerveux cérébro-spinal: sa structure, ses fonctions et ses maladies; accompagné d'un atlas de 40 planches. Baillière, Paris
Mai, JK, Forutan F (2012) Thalamus. In: Mai, J.K. and Paxinos, G (eds) The Human Nervous System, 3rd ed. Academic Press/Elsevier, San Diego, pp 620-679
Malone E (1910) Über die Kerne des menschlichen Diencephalon. Abh. d. preuß. Akad. d. Wiss.
Meynert T (1872) Vom Gehirne der Säugethiere. In: Handbuch der Lehre von den Geweben des Menschen und der Thiere, ed. S Stricker, Vol. 2, Engelmann, Leipzig
Monakow Cv (1895) Experimentelle und pathologisch-anatomische Untersuchungen über die Haubenregion, den Sehhügel und die Regio subthalamica, nebst Beiträgen zur Kenntniss früh erworbener Gross- und Kleinhirndefecte. Archiv für Psychiatrie XXVII. 1, 1-126
Münzer E, Wiener H (1902) Das Zwischen- und Mittelhirn des Kaninchens und die Beziehungen dieser Teile zum übrigen Centralnervensystem, mit besonderer Berücksichtigung der Pyramidenbahn und Schleife. Mschr Psychiat Neurol 12 (Suppl 1): 241–259
Niimi K (1949) Zur vergleichenden Cytoarchitektonik des vorderen, medianen und medialen Kernes des Sehhügels des Menschen. Acta Seh. med. Univ. Kioto 27: 116-132
Olszewski J (1952) The Thalamus of the Macaca mulatta. An Atlas for Use with the Stereotaxic Instrument, Karger, Basel
Papez JW, Aronson LR (1934) Thalamic nuclei of pithecus (Macaces) rhesus. Arch Neurol Psychiat. 32:1-26
Percheron G (2004) Thalamus. In: Paxinos G, Mai JK (eds) The Human Nervous System. Elsevier/Academic Press, San Diego, CA, pp 439-468
Rioch DM (1929) Studies on the diencephalon of carnivora: Part I. The nuclear configuration of the thalamus, epithalamus and hypothalamus of the dog and the cat. J Comp Neurol 49:1–119
Rose M (1935) Das Zwischenhirn des Kaninchens. Mem Acad Polon Sci Ser B, 1–108
Sachs E (1909) On the structure and functional relations of the optic thalamus. Brain 32:7–186
Sheps JG (1945) The nuclear configuration and cortical connections of the human thalamus. J Comp Neurol 83:1–56
Toncray JE, Krieg WJS (1946) The nuclei of the human thalamus: a comparative approach. J Comp Neurol 85:421–459
Tschisch v W (1896) Untersuchungen zur Anatomie der Großhirnganglien des Menschen. Ber d Sächs Ges d Wiss. 38 (cit from Vogt and Vogt, 1941)
Van Buren JM, Borke RC (1972) Variations and Connections of the Human Thalamus. Springer, Berlin
Vicq' d'Azyr M (1796) Traite d'anatomie et de physiologie. Paris, I. Bd. Planches anatomiques Nr. I. Cerveau de l'homme. Planche III.
Vogt C (1909) La myéloarchitecture du thalamus du cercopithèque. J Psychol Neurol (Leipzig) 12:285-324
Vogt C, Vogt O (1902) Zur Erforschung der Hirnfaserung, mit Atlas. Neuro-Biol. Arb. I, Ser. I. Verlag: Jena, Gustav Fischer, 1902

Vogt C, Vogt O (1941/1942) Thalamusstudien I-II, J Psychol Neurol (Leipzig) 50:3-74
Vogt C, Vogt O (1942) Morphologische Gestaltungen unter normalen und pathogenen Bedingungen. J Psychol Neurol 50:161–524
Walker AE (1938) The Primate Thalamus. Chicago, University Press Chicago

Supplementary Table 2. Terminology of the major nuclei and the subdivisions of the human thalamus

Hassler 1959; 1977	Feremutsch & Simma 1971	Hopf 1971	Van Buren & Borke 1972	Hirai & Jones 1989; Jones, 1990, 1998	Percheron 2004	Morel 2007	Ilinsky et al., 2018	Ding et al., 2016	TNA (2017)	Mai, Majtanik, 2018
**Envelope** (Involucrum mediale)	**F.IIa Formatio intralamellaris**	**Hüllgebiet**	**III A Intralaminar region**	**Intralaminar group** (internal medullary lamina)	**Allothalamus** (Involucrum) Formatio intralaminaris-limitans	**Medial group** (IL,MD, Midline nuclei)		**AILN Intralaminar nuclear complex**	**Intralaminar nuclei**	**Intralaminar formation (region)**
**iLa Ncl. intralaminaris anterior**	**Ncl. intralamellaris**			**Anterior intralaminar group**				**Anterior group**	**Anterior group of intralaminar nuclei**	**ILA Anterior group**
Fa Ncl. fasciculosus	N.fa Ncl. fasciculosus	Fa Ncl. fasciculosus	Fa Ncl. fasciculosus	part of medioventral ncl.				Fa fasciculosus ncl.		Fa fasciculosus ncl.
Co Ncl. commissuralis (Ncl. centralis medialis)	N.ila.c.m pars centralis medialis	Co + med part of Pm	Co Ncl. commissuralis	CeM central medial ncl. Rh rhomboid ncl. CL post central lateral ncl.	med. part of CeM	CeM MV ?	CeM Cdc central densicellular ncl.	CeM central medial ncl.	CM central medial ncl.	CeM central medial ncl. dorsal part of Re,
La Ncll. intralamellares (oralis, intermedius, caudalis)	N.ila.c.l pars centralis lateralis		iLa N. intralamellaris (CeL + PC)	CL + Pc central lateral ncl., paracentral ncl.	Ilo Situs intra-laminaris oralis		Cln central lateral ncl.	CL central lateral ncl. (m,l,d,c divisions)	CL central lateral ncl.	CeL central lateral ncl.
	N.ila.pc pars paracentralis						PC paracentral ncl.	PC paracentral ncl.	Pc paracentral ncl.	PCe paracentral ncl.
Cu Ncl. cucullaris		Cu Ncl. cucullaris	iLrc ? Ncl. intralamellaris pars orodorsalis	dorsomedial CL	CL	CL	Cs central superior ncl.	CD central dorsal ncl.		Cuc cucullar ncl.
**iLm Ncl. intralaminaris medialis**				**Posterior intralaminar group**	**Formatio or R. centralis C**			**Posterior group**	**Central group of intralaminar nuclei**	**ILC Central group**
Ce Ncl. centralis thalami (pc, mc)	N.ce Ncl. centralis	Ce Ncl. centralis (Ce.mc; Ce.pc)	Ce Ncl. centralis	CM centre médian, part CM/Pf	CPf ncl. centralis medius	CM	CM/Pf centromedian/ parafascicular ncl.	CM centromedian ncl. (l,m)	CMn centromedian ncl.	CM Ncl. centrum medianum (CMmc, CMpc)
Pf Ncl. parafascicularis	N.pf Ncl. parafascicularis	Pf	Pf Ncl. parafascicularis	Pf parafascicular ncl.	CPf ncl. centralis parafascicularis	Pf		Pf parafascicular ncl. (l,m)	Pf parafascicular ncl.	PF parafascicular ncl.
V.c.pc.i (part) Ncl. ventro-caudalis parvocellularis internus				SM submedial ncl.	sPf	sPf		SPf / RPf subparafasci-cular ncl./ retroparafasc. ncl.	SPf subparafascicular ncl. SM submedial ncl.	SM Ncl. submedius SPf subparafascicular ncl.
**iLc Ncl. intralaminaris caudalis**		**iLa.m.c Ncl. intralamellaris medialis, pars caudalis**		**Posterior group**			**LSG limitans/ suprageniculate ncl.**	**LSG limitans/supra-geniculatus**	**Posterior group of intralaminar nuclei**	**ILP Posterior group**
Li Ncl. limitans Li.m Ncl. limitans med.	N.li Ncl. limitans	Li Ncl. limitans	Li Ncl. limitans Li.m Ncl. limitans med.	Li/Sg limitans/ suprageniculate ncl. part of CL post. to MD	Li	Posterior complex:Li, Sg, Po posterior ncl.	Li limitans ncl.	Po posterior ncl.	Lim limitans ncl.	Lim Ncl. limitans LimM Ncl. limitans medialis
Ncl. suprageniculatus									SG suprageniculate ncl.	SGe suprageniculate ncl.
Li.opt Ncl. limitans opticus		Li.opt Li pars optica	Li.opt	Li						
Li.por Ncl. limitans portae		Li.por Li pars portae	Li.por	SG supragenicule ncl. Po posterior ncl. VMpo ventromedial posterior ncl.	Regio basalis	Po posterior ncl				VPb Ncl. ventroposterior basalis portae
s.Hb Ncl. subhabenularis		sHb	Hb Ncl. habenularis	-	sPf ?	sPf				SubH subhabenular ncl. ?
**Central gray** (Substantia grisea centralis thalamica; Thalamic midline nuclei)	**F.Pv Formatio paraventricularis**	**Thalamus Höhlengrau**	**I Midline** (and epithalamic region)	**Medial group (in part)**	**Allothalamus** (Involucrum) Formatio paramediana	**Medial group: midline nuclei**	**MI midline ncll.**	**Midline nuclear complex**	**Periventricular nuclei (Midline nuclei)**	**Periventricular formation and Midline nuclei**

Pm. (o,pr, c)<br>Ncl. parvomedianum (oralis, principalis, caudalis)	N.pm<br>Ncl. paramedianus	Pm<br>Ncl. paramedianus principalis		Pm. a<br>Pm. p	PV<br>paraventricular nuclei	PV<br>paraventricular nuclei	Pac<br>paraventricular nuclei	PeVA<br>periventricular area		PeV<br>periventricular ncl.
Pt.<br>Ncl. parataenialis (parvocellularis, interstitialis)	N.pt<br>Ncl. parataenialis	**Pt (o, ist)**	Pt<br>Ncl. parataenialis	Pt	Pt				PT<br>paratenial ncl.	PT<br>parataenial ncl.
			Pm<br>Ncl. paramedianus (a,p)		PV<br>paraventricular nuclei	PV<br>paraventricular nuclei	Pac<br>paraventricular nuclei	PeVA<br>periventricular area	PV<br>paraventricular ncl.	PV<br>paraventricular ncl.
Edy<br>Ncl. endymalis<br>(Ncl. reuniens)	N.re<br>Ncl. reuniens<br>N.rh<br>Ncl. rhomboidalis	Edy<br>Ncl. endymalis	Edy<br>Ncl. endymalis<br>(Ncl.reuniens, Ncl.submedius?)	MV (Re)<br>medioventral ncl. (reuniens ncl.)			Re	Re<br>reuniens ncl.<br>(medioventral ncl.)<br>Rh<br>rhomboid (central) ncl.	MV<br>medioventral ncl.<br>(Re Ncl. reuniens)	Re<br>reuniens ncl.
**Anterior nuclear group**<br>(Territorium anterius: A)	**F.A.<br>Formatio anterior**	**Vorderes Kerngebiet**	**II Anterodorsal region**	**Anterior group**	**Regio superior**	**Anterior group**		**ANC<br>Anterior nuclear complex of thalamus**	**Anterior nuclei of thalamus**	**Anterodorsal region**
A. pr<br>Ncl. anterior principalis	N.av	Apr	Apr	AV<br>anteroventral ncl.	A<br>Ncl. anterior	AV		AV	AV<br>anteroventral ncl.	AV<br>anteroventral ncl.
A. m<br>Ncl. anteromedialis	N.am			AM<br>anteromedial ncl.		(AM)		AM	AM<br>anteromedial ncl.	AM<br>anteromedial ncl.
A. d<br>Ncl. anterodorsalis	N.ad	Ad	Ad	AD<br>anterodorsal ncl.		AD		AD	AD<br>anterodorsal ncl.	AD<br>anterodorsal ncl.
D. s<br>Ncl. dorsalis superficalis	D. sf.<br>Ncl. lateralis dorsalis		D. sf.<br>Ncl. dorsalis superficalis	LD<br>lateral dorsal ncl.	S<br>Ncl. superficialis	LD	LD<br>laterodorsal ncl.	LD	LD<br>laterodorsal ncl.<br>(DSF<br>dorsal superficial ncl.)	DSF<br>dorsal superficial ncl.
**Medial nuclear region**<br>(Territorium mediale: M)	**F.M<br>Formatio medialis**	**Mediales Kerngebiet**	**III B Medial region**	**Medial group (in part)**	**Superregio medioposterio r: Regio medialis**	**Medial group**		**MNC<br>Medial nuclear complex of thalamus**	**Medial nuclei of thalamus**	**Medial region**
M<br>Ncl. medialis dorsalis or dorsomedialis	N.m<br>Ncl. medialis	Ncl. dorsomedialis	M<br>Ncl. medialis	MD<br>mediodorsal ncl.		MD	MD<br>mediodorsal ncl.	MD	MD<br>mediodorsal ncl.<br>(dorsomedial ncl.)	MD<br>mediodorsal ncl.
M. fi.<br>Ncl. medialis fibrosus	N.m.fi<br>pars fibrosa	M. fi<br>M. pars fibrosa	M	MD. mc<br>magnocellular division	M	MD. mc<br>magnocellular or medial div.		MDm<br>mc / medial division	MDmc<br>magnocellular ncl.	MDM<br>medial mediodorsal ncl.
M. fa.<br>Ncl. medialis fasciculosus	N.m.fa<br>pars fasciculosa	M. fa<br>M. pars fasciculosa		MD.l<br>lateral division		MD. pc<br>parvocell. div.		MDc<br>parvocellular (central) div.	MDpc<br>parvocellular ncl.	MDC<br>central mediodorsal ncl.<br>MDL<br>lateral mediodorsal ncl.
M. c.<br>Ncl. medialis caudalis (i,e)	N.m.c<br>pars caudalis	M. c<br>M. pars caudalis		MD.V<br>ventral division.				MDl<br>multiform lateral div.		
M. pl<br>Ncl. medialis paralamellaris	N.m.pl<br>pars paralamellaris	M. pl<br>M. pars paralamellaris		part of central lateral ncl. (CL)		MD. pl<br>pl.division, part of CL	MD/CL<br>mediodorsal/ centrolateral division	MDd<br>densocellular / paralamellar div.	MDpl<br>paralaminar part	
								MDam<br>anteromedial large-celled island		
**Lateral nuclear region**	**F.L<br>Formatio lateralis**	**L<br>Laterales Kerngebiet**	**IV Lateroventral region**	**Ventral nuclei**	**Regio lateralis**	**Lateral group**	**VLN<br>Ventral group of lateral nucleus**	**Lateral nuclear complex**<br>(dorsal group: see LP and Pul);	**Lateral nuclei of thalamus**	**Lateral region**

								ventral group		
**Oral segment of the lateral nuclear region**	**N.v.a<br>Ncl. ventralis anterior**	**V<br>Ncl. ventrooralis pars anterior**	**L.po<br>lateropolaris-fascicularis complex**	**VA**	**LR<br>Subregio lateralis rostralis**	**VA<br>ventral anterior ncl.**	**VA<br>ventral anterior ncl.**	**VA<br>ventral anterior ncl. of thalamus**	**VA<br>ventral anterior ncl.**	**VA<br>ventroanterior ncl.**
L.po (mc)<br>Ncl. lateropolaris<br>(magnocellularis)<br><br>D.o.i in part	Rt.po<br><br>VA	L.po<br>L.po.mc<br>L.am;<br><br>part of D<br>(Dorsalkerne)	L.po<br>Ncl. lateropolaris<br>[D.a,<br>V.o.anterior part],	VAmc<br>magnocellular division<br>(part of CeM)<br><br>parts of VA	VAmc<br>perifascicularis<br>(=VOM = Voi)	VAmc	VAn<br>nigral afferent zone	VAmc<br>magnocellular division	VAmc<br>VA magnocellular division	VAM<br>medial ventroanterior ncl.<br>VAmc<br>magnocellular ventroanterior ncl.
V.o.i<br>Ncl. ventro-oralis internus<br><br>D.o.i in part		V. o. i.<br>Ncl. ventro-oralis pars interna		VA<br><br>VLp<br>anterior medial part	VA<br>Ncl. ventralis anterior<br>(LR lateralis rostralis)<br>Situs polaris<br>VApo (= LPo = polar VA)	VApc<br>ventral anterior parvocellular ncl.		VApr<br>parvocellular division	VAp<br>VA principal division	
L.po.b<br>Ncl. lateropolaris basalis<br>(V.o.a)	VOM ?	L.po.b<br>pars basialis	Fa<br>Ncl. fasciculosus	VMp<br>principal ventral medial ncl.		VM<br>ventral medial ncl.	VM			VAMb<br>medial ventroanterior ncl., basal part<br>(entry zone of nigral fibers)
**O<br>Ncll. orales**	**N.v.o<br>N. ventrooralis<br>(a,m,p)**	**V<br>Ncl. ventrooralis pars interna**	**VO<br>N. ventrooralis**	**VLa<br>ventral lateral anterior ncl.**	**VO<br>Subregio lateralis oralis**	**VLp<br>ventral lateral ncl. ant. part**	**VAp<br>pallidal afferent zone**	**VL<br>ventral lateral ncl., (rostral division)**	**VL<br>ventral lateral complex<br>(anterior part)**	**VAL<br>lateral ventroanterior ncl.**
L. po. l<br>Ncl. lateropolaris lateralis<br>D.o.e<br>Ncl. dorso-oralis externus<br>(D.o.i in part)	DA<br><br>VA	La.po<br>pars parvocellularis<br>part of D<br>(Dorsalkerne)	Lpo<br>Ncl. lateropolaris<br><br>Do<br>Ncl. dorso-oralis	part of VA<br><br>VLa<br><br>+ part of VLp	VO<br>Ncl. ventralis oralis	VA<br><br><br>VLp	VAp			VAL<br>lateral ventralanterior ncl.<br>= VApr<br>principal ventroanterior ncl.
V.o.a.<br>Ncl. ventro-oralis anterior	VOA	V .o. a<br>Ncl. ventro-oralis, pars anterior	V.o.e<br>Ncl. ventro-oralis externus			VLa<br>(VAp)<br><br>VM und VA		VLR<br>rostral division	VLa<br>anterior ventrolateral ncl.	
V.o.p<br>Ncl. ventro-oralis posterior	DA	V .o<br>Ncl. ventro-oralis	V.o.i<br>Ncl. ventro-oralis internus, anterior part		VLM / VImM	VLpd				.VLA<br>anterior ventrolateral ncl.<br>(medial oral division, VLM)
V.o.m.<br>Ncl. ventro-oralis medialis	VOM	V.o.m. (?)<br>Ncl. ventro-oralis medialis<br>(pars ventralis)		VM<br>Ncl. ventralis medialis	VOM	VM		VM<br>VMb	VM<br>ventral medial complex:<br>VMb<br>basal ventromedial ncl.<br>VM<br>principal ventromedial ncl.	VALb<br>basal ventralanterior ncl.<br>(entry zone of pallidal fibers)
**Intermediate segment of the lateral nuclear mass**	**N.v.im<br>Ncl. ventralis intermedius**	**V.im<br>Ncl. ventro-intermedius**	**Ventro-intermedius**	**VLp<br>ventral posterior lateral ncl.**	**LI<br>Subregio intermedia**	**VLp<br>ventral lateral ncl. post. part**	**VL<br>cerebellar afferent territory**	**VL<br>ventral lateral ncl.**	**VL<br>ventral lateral complex<br>(posterior part)**	**VL<br>ventrolateral ncl.**
D.o.i<br>Ncl. dorso-oralis internus	DA<br>DP	part of D<br>(Dorsalkerne)	D.o.<br>Ncl. dorso-oralis	parts of VA<br>part of VLa	VA<br>Ncl. ventralis anterior	VLp<br>part of VLpl	VLd<br>VLv<br>(ventral lateral dorsal,<br>ventral lateral ventral divisions)		VLp<br>posterior ventrolateral ncl.	VAL – VLA-transition
D.im (i, e)<br>Ncl. dorso-intermedius externus, internus, superior, magnocell.	DP	D.im<br>Ncl. dorso-intermedius		VLp<br>(postero-)dorsal parts	Situs dorsalis<br>VIMps = DIps= VLps<br>Situs postremus			VLC<br>caudal division<br>(d,v,m dorsal, ventral, medial<br>[area x]<br>subdivision)		VLP<br>posterior ventrolateral ncl.<br>(lateral caudal division, VML)
V.o.i.<br>Ncl. ventro-oralis internus	VA<br>DA		V. o. i.<br>Ncl. ventro-oralis internus	VLp<br>anteromedial part	VA					
V. im. e<br>Ncl. ventromedius externus	V. im.<br>part of VOP	V. im. e.<br>Ncl. ventromedius externus	V. im. e<br>Ncl. ventro-intermedius externus	VLp<br>ventral parts	VImL / VLL /	VLa<br>^^VLpl				VLp ?

V. im. i<br>Ncl. ventro-intermedius internus	VOM<br>part of VOP	V. im. i<br>Ncl. ventro-intermedius internus	V. im. i<br>Ncl. ventro-intermedius internus		VImM / VLM	VLpd<br>VLpv				
V.c.pc.e<br>Ncl. ventro-caudalis parvocellularis externus			V.c.v<br>V.c.pc.e	VPI<br>„basalis lateralis“<br>VPI<br>ventral posterior inferior ncl.	BL<br>Ncl. basalis lateralis<br>BM<br>Ncl. basalis medialis	VPI<br>(VLPv)	VPI<br>ventral posterior inferior ncl.	VPI	VPI<br>ventral posterior inferior ncl.	VLb<br>basal ventrlateral ncl.<br>(entry zone of cerebellar fibers)
**Caudal segment of the lateral nuclear region**	**N.v.p<br>Ncl. ventral posterior<br>(l., m., acc.)**	**V.c<br>Ncl. ventro-caudalis**	**V.c<br>Ncl. ventro-caudalis<br>(Ncl. ventralis posterior)**		**Subregio lat. caud., VC;<br>Regio arcuata, Regio basalis**	**VP<br>ventroposterior complex**	**VP ventral posterior ncl.**	**VPT ventral posterior nucleus**	**VP<br>ventroposterior complex;<br>ventrobasal complex**	**VP<br>ventroposterior nci. (complex)**
V.c.e<br>Ncl. ventro-caudalis externus	VPL	V.c.e<br>Ncl. ventro-caudalis pars externa	V.c.e<br>Ncl. ventro-caudalis externus	VPL<br>ventral posterior lateral ncl.	VPL<br>Ncl. ventralis posterior	VPL	VPl<br>ventral posterior lateral nucleus	VPL<br>ventral posterior lateral nucleus	VPL<br>ventral posterolateral ncl.	VPL<br>lateral ventroposterior ncl. (Ncl. ventralis postero-lateralis
V.c.a.e<br>Ncl. ventro-caudalis anterior externus				VPLa<br>anterodorsal division of VPL	VPO<br>Ncl. ventralis posterior oralis	VPLa		VPL<br>rostral division of VPL	VPLa<br>ventral posterolateral ncl., anterior part	VPS<br>superior ventroposterior ncl<br>VPLa<br>lateral ventroposterior ncl. anterior part
V.c.p.e<br>Ncl. ventro-caudalis post. externus				VPLp<br>posterior division of VPL	VPL<br>Ncl. ventralis posterior lateralis	VPLp		VPL<br>caudal division of VPL	VPLp<br>ventral posterolateral ncl., posterior part	VPLp<br>lateral ventroposterior ncl., posterior part
V. c. i.<br>Ncl. ventro-caudalis internus,<br>V.c.a.i<br>Ncl. ventro-caudalis ant. int.	VPM	V. c. i.<br>Ncl. ventro-caudalis pars interna	V. c. i.<br>Ncl. ventro-caudalis internus	VPM<br>ventral posterior medial ncl.,<br>VPMa<br>anterior division of VPM	VPM<br>Ncl. ventralis posterior medialis	VPM	VPm<br>ventral posterior medial ncl., dorsal part	VPM<br>ventral posterior medial ncl.	VPM<br>ventral posteromedial ncl.	VPM<br>medial ventroposterior ncl.<br>(ventral posteromedial ncl.)
V.c.pc.i<br>Ncl. ventro-caudalis parvocellularis internus	N.v.p.i<br>Ncl. ventralis posterior inferior	V.c.pc.i<br>pars parvocellularis imterna	V.c.pc.<br>Ncl. ventro-caudalis parvocellularis	VMb (+ Sm)<br>basal ventral medial ncl.<br>(+ submedial ncl.)<br>(VPMpc)	VArc<br>Subregio arcuata<br>(VPMpc)	VPMpc,<br>sPF<br>subparafascicular ncl.	VPm<br>ventral posterior medial ncl., ventral part	VPMpc<br>parvocellular division of VPM<br>VMb<br>basal ventral medial ncl.	VPMpc<br>ventral posteromedial ncl., parvocellular part	VPMpc<br>medial ventroposterior ncl. parvocellular part,<br>sPF<br>Ncl. subparafascicularis
(V.c.pc.e<br>see above)		V.c.pc.e<br>pars parvocellularis externa	V.c.v<br>V.c.pc.e	VPI<br>ventral posterior inferior ncl.		VPI<br>(VLPv)	VPI<br>ventral posterior inferior ncl.	VM<br>ventral medial ncl.	VPI<br>ventral posterior inferior ncl.	VLb<br>basal ventrolateral ncl.<br>(entry zone of cerebellar fibers)
V.c.por<br>Ncl. ventrocaudalis portae	P	V.c.por<br>pars portae	Pu. o<br>pulvinar oralis	Pla<br>anterior pulvinar ncl.	PuO	Pu. A.				APUL<br>Ncl. pulvinaris anterioris
Li.por.<br>Ncl. limitans portae	P<br>Ncl. posterior	V.c.por ?<br>Ncl. ventro-caudalis portae	V.c.v.<br>Ncl. ventro-caudalis ventralis	Po (+SG)<br>suprageniculate and posterior complex	BN Ncl. basalis nodalis = Po	Po<br>posterior complex (+ SG)		Po<br>posterior ncl.	Vmpo<br>ventromedial posterior ncl.	VPb<br>basal ventroposterior ncl.<br>(entry zone of sensory fibers)
	**F.p<br>Formatio posterior)**	**Pulvinar**	**Posterior region**<br>(Pu, G l, G m, pr G)	**Lateral posterior/ Pulvinar group**	**Superregio posterior: Pulvinar**	**Posterior group**	**Pulvinar**	**DLN<br>Lateral nuclear complex: dorsal group**	**Posterior nuclei of thalamus and Pulvinar nuclei**	**Posterior region**
D. c<br>Ncl. caudalis	N.d<br>Ncl. dorsalis<br>(a., p., sf)	D	D.c<br>Ncl. dorsocaudalis	LP<br>lateral posterior ncl.	LP	-		LP<br>lateral posterior ncl.		ADPul<br>Ncl. pulvinaris anterodorsalis
Pu. sf<br>Ncl. pulvinaris superficialis	N.pu.sf<br>Ncl. pulvinaris superficialis	Pu. sf		Plim<br>medial division of inferior pulvinar ncl.,<br>parts of LD/LP/Plm						SFPul<br>Ncl. pulvinaris superficialis
Pu. o.(l,m,v)<br>Ncl. pulvinaris oralis<br>(lateralis, medialis, ventralis)		Pu. o. (l,m)<br>pars lateralis,<br>pars medialis	PuO	mostly LP and dorsal parts of anterior pulvinar ncl.	PuO (Pul.o)<br>Situs oralis;<br>PuOD (DC),<br>Ncl. lateralis posterior	LP<br>Pu A		LP<br>Pulr<br>anterior ncl. of pulvinar	LP<br>lateral posterior ncl.<br>anterior pulvinar ncl	APul<br>Ncl. pulvinaris anterior,

Pu. m. (i,d.z,v)<br>Ncl. pulvinaris medialis<br>(internus, dorsalis, zentralis,<br>ventralis)	N.pu.m<br>Ncl. pulvinaris<br>medialis	Pu. m.	PuM	Plm<br>medial pulvinar ncl.	PuM (Pul.m)<br>Situs oralis	PuM		Pulm<br>medial ncl. of<br>pulvinar	medial pulvinar ncl.	MPul<br>Ncl. pulvinaris medialis
Pu. l (s)<br>Ncl. pulvinaris lateralis<br>(superior)	Pu.l<br>Ncl. pulvinaris<br>lateralis	Pu.l.s. + Pu.l.inf.	PuL	Pll<br>lateral pulvinar ncl.	PuL (Pul.l)<br>Situs lateralis	PuL		Pull<br>lateral ncl. of<br>pulvinar	lateral pulvinar ncl.	LPul<br>Ncl. pulvinaris lateralis
Pu. l.if<br>Ncl. pulvinaris lateralis<br>inferior				Pli<br>inferior pulvinar ncl.				Puli<br>inferior ncl. of<br>pulvinar	inferior pulvinar ncl.	
Pu.(v) ig<br>Ncl. pulvinaris<br>intergeniculatus	N.pu.ig<br>Ncl. pulvinaris<br>intergeniculatus	Pu. v.	Pu.i		Ncl.<br>intergeniculatus	PuI				IGPul<br>Ncl. pulvinaris intergenicularis
Pu. v<br>Ncl. pulvinaris ventralis	-	-			Pli	-				IPul<br>Ncl. pulvinaris inferior
Pu. sb<br>Ncl. pulvinaris suprabrachialis		-			-	-				-
**Metathalamus**	**G.g<br>Formatio<br>geniculata**	**Corpus<br>geniculatum**			**G Regio<br>geniculata**	**Geniculate<br>nuclei**		**PoN<br>Posterior<br>nuclear<br>complex**	**Geniculate nuclei,<br>Metathalamus**	**Metathalamus**
**G. l.<br>Corpus geniculatum laterale**	**N.g.l<br>Ncl. geniculatus<br>lateralis**	**G.l<br>Cp. geniculatum<br>laterale**	**G l<br>Cp. geniculatum<br>laterale**	**GLD<br>Lateral geniculate body**	**GL<br>Ncl.<br>geniculatus<br>lateralis (GLd)**		**LG<br>lateral<br>geniculate ncl.**	**LG<br>lateral<br>geniculate ncl.**		**LG lateral geniculate<br>ncl.**
				LG dorsal lateral<br>geniculate ncl.				DLG dorsal<br>lateral<br>geniculate ncl.	dorsal lateral geniculate ncl.	LGD dorsal lateral geniculate<br>ncl.
G. l. mc<br>Corpus geniculatum lat.<br>magnocellularis	N.g.l.mc<br>pars<br>magnocellularis				LGd	LGN		mc	magnocellular layers	LGmc<br>dorsal lateral geniculate ncl.,<br>magnocellular part
G. l. pc<br>Corpus geniculatum lat.<br>parvocellularis	N.g.l.mde<br>pars<br>mediocellularis							pc<br>s layer<br>kc	parvocellular layers<br>koniocellular layers	LGpc<br>dorsal lateral geniculate ncl.,<br>parvocellular part
pG<br>Ncl. praegeniculatus (griseus,<br>fibrosus)	N.prg<br>Ncl.<br>praegeniculatus		PrG<br><br>pr G	Pg<br>pregeniculate ncl.	PrG Ncl.<br>praegeniculatus<br>or<br>peripedunculari<br>s	Pg		PG<br>pregeniculate<br>ncl.	ventral principal ncl.,<br>dorsal ncl.,<br>medial magnocellular ncl.	PGe<br>praegeniculate ncl.
**G. m.<br>Corpus geniculatum med.**	**N.g.m<br>Ncl. geniculatus<br>medialis**	**G.M<br>Cp. geniculatum<br>mediale**	**G m**	**MG<br>medial geniculate<br>complex**	**GM<br>Ncl.<br>geniculatus<br>medialis<br>(GMpc)**	**MGN**	**MG<br>medial<br>geniculate ncl.**	**MG<br>medial<br>geniculate ncl.**	**medial geniculate ncl.**	**MG medial geniculate ncl.**
G. m. fa<br>Corpus geniculatum med.<br>fasciculosus	N.g.m.mdc<br>pars<br>mediocellularis	Gm		MGv				VMG<br>ventral medial<br>geniculate ncl.		MGV MG, ventral division
G. m. fi<br>Corpus geniculatum med.<br>fibrosus				MGd				DMG (ad, pd)<br>dorsal medial<br>geniculate ncl.		MGD MG, dorsal division
G. m. mc<br>Corpus geniculatum med.<br>magnocellularis	N.g.m.mc<br>pars<br>magnocellularis	Gm. mc	G m mc	MG mc				MMG<br>(mc/medial)<br>magnocellular<br>(medial) ncl.		MGMC MG, magnocellular<br>division
G. m. li<br>Corpus geniculatum med.<br>limitans	N.g.m.li<br>pars limitans	G. m. li						LiMG<br>limitans part of<br>medial<br>geniculate ncl.		MGLi MG, limitans division
Rt<br>Ncl. reticulatus thalami	F.Ela<br>Formatio<br>extralamellaris	Rt<br>Reticulatum<br>thalami	-	R	PTh Ncl.<br>perithalamicus	R	Rt<br>reticular ncl.			Rt<br>reticulate ncl.

Ding SL, Royall JJ, Sunkin SM, Ng L, Facer BA, Lesnar P, et al (2016) Comprehensive cellular-resolution atlas of the adult human brain. J comp Neurol Research in Systems Neuroscience 524:3127-3481
Feremutsch K (1963) Thalamus. In: Hofer H, Schültz AH, Stark D (eds) Thalamus Primatologia Handbuch der Primatenkunde, vol 2, part 2, fasc 6, Karger, Basel, pp 1-226

Feremutsch K, Simma K (1971) Anatomy of the Normal Human Thalamus. In: Dewulf A (ed) Elsevier, Amsterdam, pp 159-168
Hassler R (1977) Architectonic organization of the thalamic nuclei. In: Schaltenbrand G, Walker AE (eds) Stereotaxy of the Human Brain. Georg Thieme Verlag Stuttgart, New York
Hirai T, Jones EG (1989) A new parcellation of the human thalamus on the basis of histochemical staining, Brain Res Rev 14:1-34
Hopf A, Krieg WJS, Feremutsch K, Simma K, Macchi G (1971) Attempt at standardization of nomenclature In: Dewulf A. Anatomy of the Normal Human Thalamus. Elsevier, Amsterdam, pp 121-138
Ilinsky I, Horn A, Paul-Gilloteaux P, Gressens P, Verney C, Kultas-Ilinsky K (2018) Human motor thalamus reconstructed in 3D from continuous sagittal sections with identified subcortical afferent territories. eNeuro 13 June 2018, ENEURO.0060-18.2018; doi: 10.1523/ENEURO.0060-18
Ilinsky IA, Kultas-Ilinsky K (1987) Sagittal cytoarchitectonic maps of the Macaca mulatta thalamus with a revised nomenclature of the motor-related nuclei validated by observations on their connectivity. J Comp Neurol. 262(3):331-364
Mai JK, Majtanik M, Paxinos G (2016) Atlas of the Human Brain. 4th ed. Academic Press/Elsevier, San Diego, CA
Mai, JK, Majtanik M (2017) Human Brain in Standard MNI Space: A comprehensive pocket atlas. Academic Press/Elsevier, San Diego, CA
Morel A (2007) Stereotactic Atlas of the Human Thalamus and Basal Ganglia. CRC Press, Boca Raton, FL
Percheron G (2004) Thalamus. In: Paxinus G, Mai JK (eds) The Human Nervous System, Elsevier/Academic Press, San Diego, CA, pp 439-468
TNA (2017) Terminologia Neuroanatomica. FIPAT.library.dal.ca. Federative Programme for Anatomical Terminology
Van Buren JM, Borke RC (1972) Variations and Connections of the Human Thalamus. Springer, Berlin

Supplementary Table 3. The conditional probabilities P(i|j) for the overlap of of the clusters defined in AHB with the areas in the eight other atlases. For abbreviations see Supplementary Table 5.

Cluster	HSL		FRM		VBB		PER		MRL		ILI		DNG		HPD	
ilm	La. M.	0,26	Md	0,40	ila	0,27	Ilo	0,56	CL	0,29	MD	0,19	CL	0,29	iLa.lo	0,56
	Pu.m	0,14	Ce	0,11	Pum	0,17	mt	0,08	MDpc	**0,16**	MDCL	0,15	MD	0,23	T.mth	0,08
	Ce	0,07	Pu.M	0,74	M	0,10	M	0,07	PuM	0,10	CMPf	0,13	VAmc	0,06	M	0,07
	D.im.i	0,07			ce-pc	0,05	CMe	0,06					CMI	0,05	Ce	0,06
**A**	Apr	0,83	A	0,77	Apr	0,66	A	0,77	AV	0,65	A	0,73	AV	0,80	A.pr	0,83
			Pt.	0,08	Pt	0,09	mt	0,11	CL	0,10	MI	0,12	AM	0,12	L.po	0,06
									AM	0,04	MD	0,07				
									AD	0,02						
									MDpc	0,01						
M	M	0,62	Md	0,87	M	0,64	M	0,90	MDpc	0,56	MD	0,63	MD	0,67	M	0,73
	Pu.m	0,12	Pu.M	0,07	Pum	0,15			CL	0,23	Pul	0,19	MDm	0,27	Pu.m	0,05
	La. M.	0,09							MDmc	0,09	MDCL	0,06				
**VAM**	L.po	0,50	VA	0,45	Lpo	0,47	VA	0,37	VApc	0,42	Vap	0,43	Vapr	0,64	L.po	0,38
	D.o.i	0,15	Rt.Po.	0,21	Fa	0,06	VO	0,32	VAmc	0,16	Vld/Vlv	0,27	Vamc	0,27	V.o.a	0,17
	V.o.i	0,15	VOM	0,06	Voe	0,05	mt	0,11	VLpv	0,07	Van	0,11			V.o.i	0,16
	La. M.	0,07	Mp	0,06			VImM-VLM	0,05								
**VAL**	V.o.a	0,32	VA	0,70	Lpo	0,40	VO	0,86	VApc	0,58	Vap	0,63	VLR	0,57	V.o.a	0,33
	D.o.e	0,23	VOA	0,19	Voe	0,26			VLa	0,14	Rt	0,17	Vapr	0,23	Z.o	0,21
	V.o.p	0,10							VM	0,06	eml	0,09	R	0,06	D.o.e	0,15
	Z.o.	0,08									Vld/Vlv	0,08			L.po	0,13
	Rt.po4	0,06													H1+H2	0,07
	D.o.e	0,06														
	Lpo	0,06														
**VL**	D.im.e	0,14	VA	0,36	Do	0,49	VImM-VLM	0,62	VLpd	0,35	VldVLv	0,57	Vapr	0,21	D.o.i	0,12
	V.im.e	0,11	DA(cbl)	0,13	Vim	0,18	Pu	0,15	VLpv	0,21	Pul	0,13	VLCd	0,15	Pu.o	0,11
	D.o.e	0,07	DP(Pulv)	0,10	Voe	0,14	VO	0,09	VLa	0,15	Vap	0,07	VLCv	0,13	D.o.e	0,09
	V.im.i	0,07	DA(pallid)	0,10	Dc	0,06			VPLp	0,05	MDCL	0,06	VPLr	0,13	D.im.i	0,08
	Pu.o.l	0,07	VOA	0,09									VLCx	0,12	V.o.i	0,08
	D.o.i	0,07											VLR	0,11	D.im.e	0,08
	Z.im.e	0,06													Vo.p	0,07
	D.im.i	0,06													V.im.i	0,06
															V.im.e	0,05

VP	V.c.a.i	0,46	VPM	0,52	Vce	0,39	Cpl	0,34	VPM	0,57	Vpl/Vpm	0,86	VPM	0,72	Ce	0,47
	V.c.pc	0,23	Ce	0,31	Vim	0,21	VPM	0,32	VPl	0,18	CMPf	0,08	VPMpc	0,11	V.c.i	0,21
	V.c.i	0,11	VPl	0,07	Do	0,18	VimM-VLM	0,26	VPMpc	0,11			VPLr	0,11	iLa	0,08
	V.im.i	0,07			Vci	0,09			VLpv	0,10						
	V.im.e	0,06			Dc	0,06										
VP (VPPC/VPb)	G.m/G.Md	0,18	VPL	0,23	Vcpc	0,24	VPM	0,27	VPI	0,24	Vpl/Vpm	0,34	VPMpc	0,24	Vcpci	0,18
	V.c.pc	0,15	Pu.M	0,16	Li	0,21	CMe	0,15	Po	0,10	Vpi	0,15	Po	0,20	Li	0,15
	Ra.prl.	0,14	VPI	0,13	Pum	0,15	Varch	0,09	VPMpc	0,07	Pul	0,08	VPI	0,10	Vcpce	0,10
	L.m.	0,11	VPM	0,10	Vci	0,10	BN	0,07	SG	0,07	MGC	0,08	Pulr	0,10	V.c.a.i	0,06
	V.c.i	0,08	Ce	0,05			Cpl	0,06			eml	0,05	VPLc	0,08	V.c.p.i	0,06
	V.c.e	0,06											Zi	0,08		
	Z.i.	0,06														
P	Pu.m	0,47	Pu.L	0,48	Pul	0,36	Pu	0,76	PuM	0,54	Pul	0,80	Pulm	0,41	Pu.m	0,36
	Pu.l	0,16	Pu.M	0,38	Pum	0,35			PuL	0,13	eml	0,08	LP	0,17	Pu.lif	0,12
	Pu.ig	0,07			Dc	0,17			VPLp	0,11			Pull	0,14	Pu.m.i	0,09
									PuA	0,06			Pulr	0,10	Pu.ig	0,09
															Pu.o	0,09
															Pu.l.s	0,05
LG	G	0,87	gl	0,74	Gl	1,00	LG	0,99	LGN	0,913	LG	0,75	DLG	0,66	G.l	0,74
											eml	0,14	LG12	0,22		
**MG**	Gm	0,82	Gm	0,692	Gm-pc	0,67	MG	0,84	MGN	0,842	MG	0,87	DMG	0,34	G.m	0,82
					Ppd	0,10	VPM	0,08					MMG	0,17		
					Pum	0,06										

Supplementary Table 4: Estimated 95th percentile values of chance concordance W_{max} and chance asymmetry W_{asym} distributions for random cluster parcellations of thalamus (a). The 95th percentile values of the global W_{max} chance distribution for random parcellations of the thalamus (b). The 95th percentile values of the global asymmetry W_{max} chance distribution for random parcellations of the thalamus (c).

a)

	Regions (Cluster)									
	IL	AN	CM/PF	MD	VAM	VAL	VL	VP	P	LGB/MGB
95th percentile of W_{max}	0.41	0.45	0.47	0.39	0.44	0.52	0.51	0.48	0.35	0.25
95th percentile of W_{asym}	0.23	0.14	0.19	0.23	0.25	0.29	0.22	0.26	0.19	0.21

b)

**AHB**	0.3487	0.3870	0.4060	0.3780	0.3537	0.3656	0.4618	0.3555
	**MRL**	0.3627	0.4333	0.3993	0.3324	0.3394	0.4831	0.3342
		**HSL**	0.4745	0.4433	0.3533	0.3481	0.5218	0.3576
			**FRM**	0.3641	0.4440	0.4463	0.4104	0.4405
				**ILI**	0.4081	0.4155	0.4295	0.4079
					**VBB**	0.3320	0.4973	0.3322
						**HPD**	0.4963	0.3345
							**PER**	0.4905
								**DNG**

c)

**AHB**	0.1014	0.1765	0.2092	0.1581	0.1141	0.1305	0.3340	0.1194
	**MRL**	0.1320	0.2546	0.1984	0.0701	0.0792	0.3758	0.0670
		**HSL**	0.3291	0.2718	0.1239	0.1087	0.4454	0.1237
			**FRM**	0.1208	0.2711	0.2834	0.2104	0.2651
				**ILI**	0.2114	0.2288	0.2570	0.2121
					**VBB**	0.0709	0.3864	0.0690
						**HPD**	0.4028	0.0728
							**PER**	0.3824
								**DNG**

Supplementary Table 5: Lists of abbreviations for all atlases used in the study.

List of Abbreviations for Mai et al. (2016) and Mai and Majtanik (2017)

AD	anterodorsal nucleus
AM	anteromedial nucleus
APUL	nucleus pulvinaris anterioris
APUL	nucleus pulvinaris anterioris
AV	anteroventral nucleus
CeMe	central medial nucleus
CL	central lateral nucleus
CM	centromedian nucleus,
CMmc	centromedian nucleus, pars magnocellularis
CMpc	centromedian nucleus, pars parvocellularis
Cuc	cucullar nucleus
DPUL	nucleus pulvinaris dorsalis
DPUL	nucleus pulvinaris dorsalis
DPUL	nucleus pulvinaris dorsalis
DSF	dorsal superficial nucleus
Fa	nucleus fasciculosus
Hb	habenula
IGPUL	nucleus pulvinaris intergenicularis
IL	nucleus intralaminaris
ILA	nucleus intralaminaris, anterior group
ILC	nucleus intralaminaris, central group
ILP	nucleus intralaminaris, posterior group
IML	nucleus intralaminaris medialis
LG	nucleus geniculatus lateralis
LGmc	nucleus geniculatus lateralis, pars magnocellularis
LGpc	nucleus geniculatus lateralis, pars parvocellularis
Lim	nucleus limitans
LimM	nucleus limitans medialis
LPUL	nucleus pulvinaris lateralis
MD	mediodorsal nucleus,
MDmc	mediodorsal nucleus, magnocellular part, medial MD
MDpc	mediodorsal nucleus, parvocellular part, central MD
MDpl	paralaminar, (paralamellar, densocellular, multiforme) MD
MPUL	nucleus pulvinaris medialis
PC	paracentral nucleus
pc	posterior commissure
PF	parafascicular nucleus
PGe	nucleus praegeniculatus
PT	nucleus parataenialis
PV	paraventricular nucleus
Re	reuniens nucleus
RT	nucleus reticulatus
Rt	reticular thalalamic nucleus
SFPUL	nucleus pulvinaris superficialis
SGe	nucleus suprageniculatus
SPf	subparafascicular nucleus
VA	ventroanterior nucleus
VAL	lateral ventralanterior nucleus
VAL	lateral ventroanterior nucleus
VALb	basal ventralanterior nucleus (entry zone of pallidal fibers)
VAM	medial ventroanterior nucleus
VAMb	medial ventroanterior nucleus, basal part, (entry zone of nigral fibers)
VAmc	magnocellular ventroanterior nucleus
VApr	principal ventroanterior nucleus
VL	ventral lateral Ncl
VLb	basal ventrlateral nucleus, (entry zone of cerebellar fibers)
VLb	nucleus ventrolateralis basalis
VLl	lateral ventrolateral nucleus
VLm	medial ventrolateral nucleus
VP	ventroposterior nucleus
VPb	nucleus ventroposterior basalis portae
VPb	nucleus ventroposterioris basalis, (entry zone of sensory fibers)
VPL	
VPLa	nucleus ventroposterioris lateralis, pars anterior
VPLp	nucleus ventroposterioris lateralis, pars posterior
VPM	nucleus ventroposterioris medialis
VPMpc	nucleus ventroposterioris medialis parvocellularis

List of Abbreviations for Ding et. at (2016)

AD	anterodorsal nucleus of thalamus
AILN	anterior group of intralaminar nuclei
AM	anteromedial nucleus of thalamus
AV	anteroventral nucleus of thalamus
CD	central dorsal nucleus of thalamus
CeM	central medial nucleus of thalamus
CL	central lateral nucleus of the thalamus
CLl	lateral division of central lateral nucleus
CLm	medial division of central lateral nucleus
CM	centromedian nucleus of thalamus
CMl	lateral division of centromedian nucleus of thalamus
CMm	medial division of centromedian nucleus of thalamus
DLG	dorsal lateral geniculate nucleus
DLN	dorsal group of lateral nucleus
Fa	fasciculosus nucleus of thalamus
LD	lateral dorsal nucleus of thalamus
LG	lateral geniculate nucleus
Lim	limitans nucleus
LiMG	limitans part of medial geniculate nucleus
LP	lateral posterior nucleus of thalamus
MD	mediodorsal nucleus of thalamus
MDc	parvocellular (central) division of MD
MDd	densocelllular (paralamellar) division of MD
MDl	multiform (lateral) division of MD
MDm	magnocellular (medial) division of MD
MG	dorsal medial geniculate nucleus
PC	paracentral nucleus of thalamus
PeVA	periventricular area of thalamus
Pf	parafascicular nucleus of thalamus
Pf	parafascicular nucleus of thalamus
PG	pregeniculate nucleus
Po	posterior nucleus of thalamus
PoN	posterior nuclear complex of thalamus
Pt	parataenial nucleus of thalamus
Pul	pulvinar of thalamus
Puli	inferior nucleus of pulvinar
Pull	lateral nucleus of pulvinar
Pulm	medial nucleus of pulvinar
Pulr	anterior nucleus of pulvinar
Re	reuniens nucleus (medioventral nucleus) of thalamus
RN	red nucleus
RPf	retroparafascicular area of thalamus
SGN	suprageniculate nucleus of thalamus
SPf	subparafascicular nucleus of thalamus
VA	ventral anterior nucleus of thalamus
VAmc	magnocellular division of VA
VApr	parvocellular division of VA
VL	ventral lateral nucleus of thalamus
VLC	caudal division of VL
VLCd	dorsal subdivision of VLC
VLCv	ventral subdivision of VLC
VLCx	medial subdivision of VLC (area x)
VLN	ventral group of lateral nucleus
VLR	rostral division of VL
VM	ventral medial nucleus of thalamus
VMb	basal ventral medial nucleus
VMG	ventral medial geniculate nucleus
VPI	ventral posterior inferior nucleus
VPL	ventral posterior lateral nucleus
VPLc	caudal division of ventral posterior lateral nucleus
VPLr	rostral division of ventral posterior lateral nucleus
VPM	ventral posterior medial nucleus
VPMpc	parvocellular division of VPM
VPT	ventral posterior nucleus of thalamus

List of Abbreviations for Morel (2007)

ac	Anterior commissure
AD	Anterodorsal nucleus
AM	Anteromedial nucleus

AV	Anteroventral nucleus
CeM	Central medial nucleus
CL(p)	Central lateral nucleus (posterior part)
CM	Centre médian nucleus (or centromedian)
Hb	(l,m) Habenular nucleus (lateral and medial subdivisions)
LD	Lateral dorsal nucleus
LGN	(mc) Lateral geniculate nucleus (magnocellular layers)
Li	Limitans nucleus
LP	Lateral posterior nucleus
MD pc	Mediodorsal nucleus (parvocellular division)
MDmc	Mediodorsal nucleus (magnocellular division)
MGN	Medial geniculate nucleus
PC	Posterior commissure
Pf	Parafascicular nucleus
Po	Posterior nucleus
PuA	Anterior pulvinar
PuI	Inferior pulvinar
PuL	Lateral pulvinar
PuM	Medial pulvinar
Pv	Paraventricular nuclei
SG	Suprageniculate nucleus
sPf	Subparafascicular nucleus
VAmc	Ventral anterior nucleus (magnocellular division)
VApc	Ventral anterior nucleus parvocellular division)
VLa	Ventral lateral anterior nucleus
VLpd	Ventral lateral posterior nucleus (dorsal division)
VLpv	Ventral lateral posterior nucleus (ventral division)
VM	Ventral medial nucleus
VPI	Ventral posterior inferior nucleus
VPL(a)	Ventral posterior lateral nucleus (anterior division)
VPL(p)	Ventral posterior lateral nucleus (posterior division)
VPM	Ventral posterior medial nucleus
VPMpc	Ventral posterior medial nucleus, parvocellular division

List of Abbreviations for Van Buren and Borke (1971)

Ad	nuleus anterodorsalis
Am	nuleus anteromedialis
Apr	nuleus anteroprincipalis
Cclg	Corporis callosi genu
Cclr	Corporis callosi rostrum
Ccls	Corporis callosi splenium
C cl t	Corporis callosi truncus
Ce N.	centralis
Cemc	nuleus centralis magnocellularis
Ce pc	nuleus centralis parvocellularis
Cmp	Commissura posterior
Co	nuleus commissuralis
Dc	nuleus dorsocaudalis
Do	nuleus dorsooralis
D sf	nuleus dorsalis superficialis
Fa	nuleus fasciculosus
Gl	Corpus geniculatum laterale
Gl mc	Corpus geniculatum laterale, pars magnocellularis
G 1 pc	Corpus geniculatum laterale, pars parvocellularis
Gm	Corpus geniculatum mediale
Gm mc	Corpus geniculatum mediale, pars magnocellularis
Gm pc	Corpus geniculatum mediale, pars parvocellularis
H	Habenula
Hl	nuleus habenularis lateralis
Hm	nuleus habenularis medialis
iLa	nuleus in tralamellaris
Li	nuleus limitans
Lpo	nuleus lateropolaris
M	nuleus medialis
Pf	nuleus parafascicularis
Pm	nuleus paramedianus
Pm a	nuleus paramedianus anterior
Pm p	nuleus paramedianus posterior
pr G	nuleus praegeniculatus
Pt	nuleus parataenialis
Pu	nuleus pulvinaris
Pu i N.	pulvinaris in tergenicula tus
Pu I N.	pulvinaris lateralis
Pum N.	pulvinaris medialis
Pu o.	nuleus pulvinaris oralis
Pv N.	paraventricularis hypothalami
R N.	reticularis
S m th	Stria medullaris thalami
Vc	nuleus ventrocaudalis
V c e	nuleus ventrocaudalis externus
V ci	nuleus ventrocaudalis internus
V cpc	nuleus ventrocaudalis parvocellularis
Vcv	nuleus ventrocaudalis ventralis
Vime	nuleus ventrointermedius externus
Vimi	nuleus ventrointermedius internus
Vo e	nuleus ventrooralis externus
Voi	nuleus ventrooralis internus
Vom	nuleus ventrooralis medialis

List of Abbreviations for Hassler (1977); Hassler et al. (1979)

A.tr.W	Area triangularis Wernicke
Apr	Nucleus anterior principalis
A. pr	Nucleus anterior principalis
A. m	Nucleus anteromedialis
A. if	Nucleus anteroinferior
A. r	Nucleus anteroreuniens
A. d	Nucleus anterodorsalis
B	Anterior perforated substance
Ce.	Nucleus centralis
Cm.a.	Anterior commissure
Co	Nucleus commissuralis
Cu	Nucleus cucullaris
D.c	Nucleus ventrocaudalis
D.im.e	Nucleus dorsointermedius externus
D.im.i	Nucleus dorsointermedius internus
D.o.e	Nucleus dorsooralis externus
D.o.i	Nucleus dorsooralis internus
D.sf.	Nucleus dorsalis superficialis
Fa	Nucleus fasciculosus thalami
Fx	Fornix
G.m	Corpus geniculatum medialis
G.l	Corpus geniculatum lateralis
H.i.	Ganglion habenulae internus
H.m	Ganglion habenulae medialis
iLm	Nucleus intralaminaris medialis
iLa.o	Nucleus intralaminaris oralis
La. M.	Lamella medialis thalami
la.p.l	Lamina palidii lateralis
la.p.m	Lamina palidii medialis
Li	Nucleus limitans thalami
L.po	Nucleus lateropolaris thalami
M	Nucleus medialis
Pf.	Nucleus parafascicularis
Ppd.	Nucleus peripendicularis
prG	Praegeniculatum
Pu.ig	Nucleus pulvinaris intergeniculatus
Pu.l	Nucleus pulvinaris lateralis
Pu.m	Nucleus pulvinaris medialis
Pu.o.l	Nucleus pulvinaris orolateralis
Pu.o.m	Nucleus pulvinaris oromedialis
Pu.o.v	Nucleus pulvinaris oroventralis
Pu.sf	Nucleus pulvinaris superficialis
Pt.	Nucleus parataenialis
Pv	Nucleus paraventricularis
Ru	Nucleus ruber
Rt.po.	Nucleus reticularis polaris
Rt.pu.	Nucleus reticularis pulvinaris
st. m	Stria medullaris thalami
V.c.a.i	Nucleus ventrocaudalis anterior internus
V.c.e	Nucleus ventrocaudalis externus
V.c.i	Nucleus ventrocaudalis internus
V.c.p.e	Nucleus ventrocaudalis posterior externus
V.c.pc	Nucleus ventrocaudalis parvocellularis
V.c.pc.e	Nucleus ventrocaudalis parvocellularis externus
V.c.pc.i	Nucleus ventrocaudalis parvocellularis internus
V.c.por	Nucleus ventrocaudalis portae
V.im.e	Nucleus ventrointermedius externus
V.im.i	Nucleus ventrointermedius internus
V.o.a	Nucleus ventrooralis anterior
V.o.i	Nucleus ventrooralis internus
V.o.m	Nucleus ventrooralis medialis
V.o.p	Nucleus ventrooralis posterior
Z.c.e	Nucleus zentrocaudalis externis
Z.c.i	Nucleus zentrocaudalis internis
Z.i.	Zona incerta

Z.im	Nucleus zentrointermedius
Z.im.e	Nucleus zentrointermedius externus
Z.im.i	Nucleus zentrointermedius internus
Z.o.	Nucleus zentrooralis

List of Abbreviations for Illinsky et al. (2018)

A	Anterior Nucleus
CL	Centrolateral Nucleus
CM/Pf	Centromedian/Parafascicular
GPl	Globus Pallidus lateral
GPm	Globus Pallidus medial
Hbl	Habenula lateral
Hbm	Habenula medial
ic	Internal Capsule
LD	Laterodorsal Nucleus
LG	Lateral Geniculate Nucleus
Li	Limitans Nucleus
MB	Mamillary Body
MD	Mediodorsal Nucleus
MG	Medial geniculate
MGC	MG complex
ot	Optic tract
PC	Paracentral Nucleus
Po	Pontine Nucleus
Pul	Pulvinar
R	Red Nucleus
Rt	Reticular Nucleus
VAn	Ventral Anterior nigral Nucleus
VAp	Ventral Anterior pallidal Nucleus
VLd/VLv	Ventral Lateral dorsal/medial Nucleus
VPi	Ventral posterior inferior Nucleus
VPl/VPm	Ventral Posterior lateral/medial Nucleus

List of Abbreviations for Hopf 1971

A	Ncl. anterior principalis
Ad	Ncl. anterodorsalis
Ce.mc	Ncl. centralis thalami; centre médian (magnocellular division)
Ce.pc	Ncl. centralis thalami; centre médian (parvocellular division)
Co	Ncl. commissuralis
Cu	Ncl. cucullaris
D	Ncl. dorsalis
D. sf.	Ncl. dorsalis superficalis
D.im	Ncl. dorsomintermedius
Edy	Ncl. endymalis
Fa	Ncl. fasciculosus
G. m. li	Corpus geniculatum mediale, pars limitans
G.l	Cp. geniculatum laterale
Gm	Cp. geniculatum mediale
Gm. mc	Corpus geniculatum mediale, pars magnocellularis
Hüllgebiet	Involucrum
iLa.m (c)	Ncl. intralamellaris medialis (caudal part)
Li	Ncl. limitans
Li.opt	Ncl. limitans opticus
Li.por	Ncl. limitans portae
M. c	Ncl. medialis caudalis
M. fa	Ncl. medialis fasciculosus
M. fi	Ncl. medialis fibrosus
M. pl	Ncl. medialis paralamellaris
Pf	Ncl. parafascicularis
Pm	Ncl. parvomedianum
Pm (m)	med. part of parvomedianum
Pt (o, ist)	Ncl. parataenialis (oral, interstitial part)
Pu. m.	Ncl. pulvinaris medialis
Pu. o. l	Ncl. pulvinaris oralis (pars lateralis)
Pu. o. m.	Ncl. pulvinaris oralis (pars medialis)
Pu. sf	Ncl. pulvinaris superficialis
Pu. v.	Ncl. pulvinaris ventralis
Pu.l.inf.	Ncl. pulvinaris lateralis inferior
Pu.l.s.	Ncl. pulvinaris lateralis (superior)
Rt	Reticulatum thalami
sHb	Ncl. subhabenularis
V. c. i.	Ncl. ventro-caudalis internus
V. im. E	Ncl. ventromedius externus
V. im.i..	Ncl. ventro-intermedius internus
V.c.e	Ncl. ventro-caudalis externus
V.o.pc	Ncl. ventro-oralis parvocellularis
Z. im. e.	Ncl. zentromedius externus
Z.im	Ncl. zentro-intermedius
Z.im.i	Ncl. zentrolateralis intermedius internus
Z.o	Ncl. zentro-oralis

List of Abbreviations for Feremutsch (1963); Feremutsch & Simma (1971)

A	Ncl. anterior
Ce (CEM)	Ncl. centrum medianum (Ncl. centralis, PNA)
CGL (GL)	Corpus geniculatum laterale
CGM (GM)	Corpus geniculatum mediale
Ci	Ncl. circularis
D.sf	Ncl. dorsalis superficialis
DA (LA)	Ncl. dorsalis anterior (Ncl. lateralis anterior)
DP (LDP)	Ncl. dorsalis posterior (Ncl. lateralis, pars dorsalis posterior)
Fa	Ncl. fasciculosus
Ila.cl	Nc. intralaminaris centrolateralis
Ila.cm	Nc. intralaminaris centromedialis
L	Ncl. lateralis
LD	Ncl. lateralis, pars dorsalis
Li	Ncl. limitans
LV (VOA)	Ncl. lateralis, pars ventralis (Ncl. ventralis oralis, pars anterior)
LV (VOP)	Ncl. lateralis, pars ventralis (Ncl. ventralis oralis, pars posterior)
LVP	Ncl. lateralis, pars ventralis posterior
LVP.l (VPL)	Ncl. lateralis, pars ventralis posterior lateralis
LVP.m (VPM)	Ncl. lateralis, pars ventralis posterior medialis
M.fa	Ncl. medio-dorsalis pars fasciculosa
M.fi	Ncl. medio-dorsalis pars fibrosa
M.pl	Ncl. medio-dorsalis pars paralamellaris
MD	Ncl. medio-dorsalis
P	Ncl. posterior, part of CI
Pf	Ncl. parafascicularis
Pm	Ncl. parvomedianum (Territorium periventriculare)
Pt	Ncl. parataenialis
Pu	Ncl. pulvinaris
Pu.IG	Ncl. pulvinaris inferior (Intergeniculatum)
Pu.L	Ncl. pulvinaris lateralis
Pu.M	Ncl. pulvinaris medialis
Pu.Sf	Ncl. pulvinaris superficialis
py	Territorium periventriculare
RE	Ncl. reuniens massae intermediae
Rt (LR)	Ncl. reticularis (Ncl. lateralis, pars reticularis)
Rt.Po	Ncl. reticularis polaris
V.Im	Ncl. lateralis, pars ventralis posterior
VA (LA)	Ncl. ventralis anterior (Ncl. lateralis, pars principalis)
VOM (LA)	Ncl. ventralis oralis, pars medialis
VPI	Ncl. lateralis, pars ventralis posterior internus

List of Abbreviations for Hirai & Jones (1989); Jones (1990, 1998)

AD	anterodorsal ncl.
AM	anteromedial ncl.
AV	anteroventral ncl.
CeM.	central medial ncl.
CL	central lateral ncl.
CL post	central lateral ncl., posterior part
CM	centre médian, part CM/Pf
CM	centre médian ncl.
GLd	dorsal lateral geniculate ncl.
GLD	dorsal lateral geniculate ncl.
L/Sg	limitans/ suprageniculate ncl.
LD	lateral dorsal ncl.
Li (L)	limitans ncl.
LP	lateral posterior ncl.
MD	mediodorsal ncl.
MD. l.	mediodorsal ncl., lateral division
MD. mc	mediodorsal ncl.magnocellular division
MD. v.	mediodorsal ncl., ventral division
MG	medial geniculate complex
MG mc	medial geniculate complex, magnocellular division
MGd	medial geniculate complex, dorsal division
MGv	medial geniculate complex, ventral division
MV (Re)	medioventral ncl. (reuniens ncl.)
Pc	paracentral ncl.
Pc	paracentral ncl.
Pf	parafascicular ncl.
Pg	pregeniculate ncl.
Pla	anterior pulvinar ncl.

Pli	inferior pulvinar ncl.
Pll	lateral pulvinar ncl.
Plm	medial pulvinar ncl.
Pm. a	anterior paramedian ncl.
Pm. p	posterior paramedian ncl.
Po	posterior ncl. (Basalis „Halo“)
Pt	parataenial ncl.
R	reticular ncl.
Rh	rhomboid ncl.
Sg	suprageniculate ncl.
SG	posterior nucleus
Sm	submedial ncl.
SM	Ncl. submedius
Sm	submedial ncl.
VA	ventral anterior ncl.
VAmc	magnocellular ventral anterior ncl.
VLa	ventral lateral anterior ncl.
VLam	ventral lateral anterior ncl., medial part
VLp	ventral lateral posterior ncl.
VLp	ventral lateral posterior ncl.
VLpd	ventral lateral posterior ncl., dorsal part
VM	principal ventral medial ncl.
VMb	basal ventral medial ncl.
VMpo	(Basalis nodalis)
VMP	ventromedial posterior ncl.
VPI	ventral posterior inferior nucleus (Basalis lateralis)
VPL	ventral posterior lateral ncl.
VPLa	anterior division of ventral posterior lateral ncl.
VPLp	posterior division of ventral posterior lateral ncl.
VPM	ventral posterior medial ncl.
VPMa	anterior division of VPM
VPMpc	parvocellular division of of ventral posterior medial nucleus (VMb, Varc)
VPO	oral division of ventral posterior lateral ncl.
(VPS)	

List of Abbreviations for Percheron (2004)

A	Ncl. anterior
BN	Ncl. basalis
CeM	nucleus centralis medialis
CL	Ncl. centralis lateralis
CM	centre médian
Co	Ncl. commissuralis
LG	Ncl. geniculatus lateralis
LGd	Ncl. geniculatus lateralis, pars dorsalis
Li	Ncl. limitans
LP	Ncl. lateralis posterior
M	Regio medialis
MG	Ncl. geniculatus medialis
Pf	Ncl. parafascicularis
Pg	Ncl. praegeniculatus
Pu	Ncl. pulvinaris
PuA	Ncl. pulvinaris anterior
PuI	Ncl. pulvinaris inferior
PuIg	Ncl. pulvinaris intergeniculatus
PuL	Ncl. pulvinaris lateralis
PuM	Ncl. pulvinaris medialis
PuO	Ncl. pulvinaris oralis
PuOD	Ncl. pulvinaris oralis dorsalis
R	Ncl. reticularis
sPf	Ncl. subparafascicularis
VA	Ncl. ventralis anterior
VAmc	Ncl. ventralis anterior, pars magnocellularis
VArc	Ncl. ventralis arcuata
VIm	Ncl. ventralis intermedius
VImL	Ncl. ventralis intermedius, pars lateralis
VImM	Ncl. ventralis intermedius, pars medialis
VL	Ncl. ventralis lateralis
VLL	Ncl. ventralis lateralis, pars lateralis
VLp	Ncl. ventralis lateralis, pars posterior
VO	Ncl. ventro-oralis
VP	Subregio lateralis caudalis
VPL	Ncl. ventroposterior lateralis
VPM	Ncl. ventroposterior medialis
VPO	Ncl. ventroposterior oralis
